# Sick or rich: Assessing the selected soil properties and fertility status across the tea-growing region of Dooars, West Bengal, India

**DOI:** 10.3389/fpls.2022.1017145

**Published:** 2022-12-20

**Authors:** Harisadhan Malakar, Gagan Timsina, Jintu Dutta, Arup Borgohain, Diganta Deka, Azariah Babu, Ranjit Kumar Paul, Md. Yeasin, Feroze Hasan Rahman, Saumik Panja, Tanmoy Karak

**Affiliations:** ^1^Tocklai Tea Research Institute, Tea Research Association, Jorhat, Assam, India; ^2^North Bank Advisory Centre, Tea Research Association, Sonitpur, Assam, India; ^3^Upper Assam Advisory Centre, Tea Research Association, Dibrugarh, Assam, India; ^4^Indian Agricultural Statistics Research Institute, Indian Council of Agricultural Research, New Delhi, India; ^5^Department of Soil, Indian Council of Agricultural Research- Agricultural Technology Application Research Institute Kolkata, West Bengal, India; ^6^Environmental Health and Safety, University of California Merced, Merced, CA, United States

**Keywords:** nutrient index, organic carbon, pH, potassium, soil, sulphur, tea

## Abstract

Harnessing the potential yields of evergreen perennial crops like tea (*Camellia sinensis* L.) essentially requires the application of optimum doses of nutrients based on the soil test reports. In the present study, the soil pH, organic carbon (OC), available potassium as K_2_O (AK), and available sulphur (AS) of 7300 soil samples from 115 tea estates spread over the Dooars ranging from 88°52’E to 89°86’E longitude and 26°45’N to 27°00’N latitude of West Bengal, India have been documented. About 54% of soil samples were found within the optimum range of soil pH (4.50-5.50) for tea cultivation. The overall range of OC was found from 0.28% to 6.00% of which, 94% of the analyzed samples were within the range of satisfactory to excellent level of OC i.e. (>0.80% to 6.00%). Around 36.3% of soil samples were found to have high AK (>100 mg kg^-1^) but 37.1% of soils were found to have high AS content (>40 mg kg^-1^). The nutrient index status of soil pH was low in Dam Dim, Chulsa, Nagrakata, Binnaguri, and Jainti sub-districts. Soils from five sub-districts had a high nutrient index (2.47 to 2.83) for soil organic carbon. However, it existed in the medium index (1.69 and 2.22) for Dalgaon and Kalchini sub-districts. Only Nagrakata sub-district soil samples were in the high nutrient index (2.65) for AK. All analyzed samples showed a medium nutrient index (1.97 to 2.27) for AS. The result indicated that soil pH was significantly negatively correlated with soil OC (-0.336) and AK (-0.174). However, the soil OC was significantly positive correlated with AK (0.258) and AS (0.100). It could be concluded that a balanced fertilizer application would be needed as a part of the soil improvement program through soil chemical tests for sustainable tea cultivation.

## Introduction

Consumption of more than two billion cups of tea prepared from young shoots of tea plants (*Camellia sinensis* L.) per day by tea drinkers reflects its global popularity which is not only due to its non-alcoholic property but also for its stimulating and medicinal properties (Drew, 2019). The total global production of made tea in 2019 was 6.50 million tonnes, and India contributed around 21.4% of the total global production (FAOSTAT, 2021). In India, tea cultivation is spread over 17 states, and the North-Eastern states cover about 75% of the total tea plantation areas. According to the data from the Tea Board of India (www.teaboard.gov.in), India produced 1,390 million tonnes of tea in 2019. Out of that, 424 million kilograms were contributed by the state of West Bengal.

The Dooars is one of the major tea-growing regions in the Indian state of West Bengal which share around 32% of the total tea production of West Bengal. Tea produced from the Dooars tea-growing region is famous for black teas, viz. CTC (Curl, Tears, and Cut) and Orthodox teas (Barman et al., 2020).

The tea plant is a monoculture perennial crop and its productivity depends on several factors, of which soil health is one of the vital factors as it facilitates productivity. However, the soil’s physico-chemical properties might be changed due to prolonged tea cultivation (Chen et al., 2006). Evaluation of soil fertility status of tea growing soil of a region is essential for sustainable tea production. A recent study highlighted that indiscriminate use of chemical fertilizers affects soil health which subsequently makes the soil sick (Medhe et al., 2012; Bashagaluke et al., 2018). Therefore, periodical evaluation of soil fertility is of paramount importance for soil nutrient management and thus sustainable tea production (Sahrawat et al., 2010; Singh et al., 2018). The site-specific information on soil fertility is essential to managing the soil and maintaining tea productivity (Chandrakala et al., 2018). Karak et al. (2015) reported the physical and chemical properties of 991 surface soil samples collected from 15 large tea estates (TEs) in the tea bowl of India (Dibrugarh and Tinsukia districts) in the state of Assam. Barooah et al. (2020) conducted a study to evaluate the fertility status of tea-growing soils of five different villages in the Lahowal block of Dibrugarh district, Assam, India. However, from the literature survey, it has been found that there is no information available on the physico-chemical properties of soils in the tea-growing regions of Dooars, West Bengal, India. Soil pH influences soil’s biological, chemical, and physical properties and processes like ammonia volatilization, nitrification and denitrification, mineralization of organic matter, precipitation and dissolution of organic matter and heavy metals, biodegradation of organic pollutants, soil enzyme activities, rhizosphere processes, etc. (Neina, 2019). The soil oxidizable organic carbon (OC) plays a vital role through soil nutrient build-up (Sithole and Magwaza, 2019), increases soil infiltration, decreases soil water evaporation (Li et al., 2019), and also plays a major role in the global carbon cycle and climate change (Ramesh et al., 2019). Potassium is the second major nutrient for tea, and it is considered to play a critical role in the photosynthetic activity of plant growth and has a fundamental role in stomata opening control (Andrés et al., 2014; Tränker et al., 2018). Sulphur (S) is one of the essential elements for the growth and development of the tea plant (Karak et al., 2015) by playing role in major plant physiological activities, viz. photosynthesis, and respiration (Hawkesford and De Kok, 2006; Haneklaus et al., 2007). For the tea plant, S also plays a significant role in determining the quality parameters of tea which includes theaflavin, flavonol, glycosides, thearubigin, and flavonol glycosides (Chakravartee, 1996).

Therefore, the present study was formulated to evaluate the fertility status of the tea-growing soils of the Dooars region, West Bengal, India by adopting the nutrient index approach in which some important soil parameters, viz. soil pH, oxidizable organic carbon (OXC), available potassium (K) as K_2_O (AK) and available sulphur (AS) has been documented. This information not only helps understand the soil fertility status of the areas but will also enable the tea planters to take the appropriate corrective nutrient management measures based on analyzed data.

## Materials and methods

### Study area

The study area covers around one lakh hectares in the Dooars region which lies between 26°45’-27°00’ North latitude and 88°52’-89°86’ East longitude with elevation ranges between 90 m and 1750 m. Dooars is the gateway to the hill station of Darjeeling of West Bengal and the Sikkim in India. Geographically, this area is situated in the Himalayan foothills and has scenic beauty. This area has wildlife-rich tropical forests, and numerous hill streams cutting across the tea gardens and undulating plains. The Dooars tea growing area is divided into seven sub-districts, namely Binnaguri, Chulsa, Dalgaon, Dam Dim, Janiti, Kalchini, and Nagrakata. However, as per the district borderline, this area falls under the Jalpaiguri, Alipurduar, Cooch Behar, and part of the Kalimpong district of West Bengal state in India. Most of the Tea Estates of Nagrakata, Chulsa, and part of Dam Dim and Jainti sub-districts are in comparatively higher elevations. The rest of the tea estates are situated in undulating plains of lower elevations. The North Bengal Regional Research & Development Centre, Nagrakata Branch of Tea Research Association, Jalpaiguri, West Bengal, India looks after the tea cultivation-related research activities in the aforementioned sub-districts. To sustain their daily life, thousands of people in this region depend on the tea estates and their allied factories as well as tourism and timber.

### The climate of the study area

Dooars area is a part of the monsoon climate zone of South Eastern Asia, and generally, the monsoon starts in the middle of May and continues until the end of September. Winters are cold with foggy mornings and nights. The meteorological data were recorded at North Bengal Regional Research & Development Centre, Tea Research Association, Nagrakata, Jalpaiguri, West Bengal. Forty years of long-term data revealed that the average maximum and minimum temperatures were 28.81°C and 18.65°C, respectively. The average daily rainfall was recorded as 10.34 mm and annual rainfall was 3777 mm. The average morning and evening humidity (%) were 91.51 and 64.18, respectively. The average sunshine hour was recorded as 5.72 h.

### Soil sampling, pretreatment, and analysis

**Figure 1:**
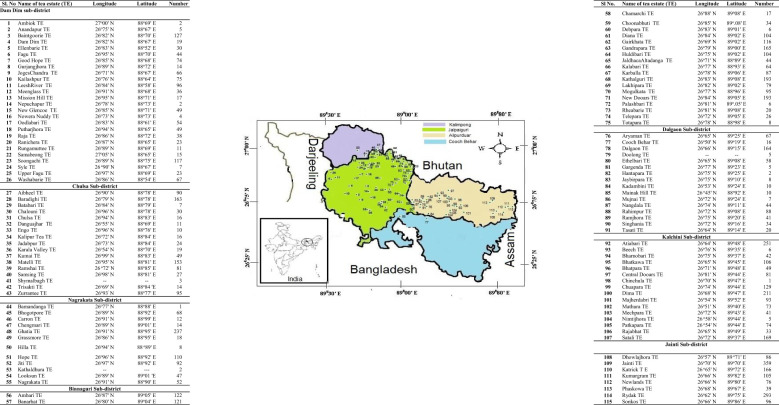
Details of 7300 soil samples collected from 115 tea estates spread over seven sub-districts of Dooars, West Bengal, India.

Out of 183 tea estates (TEs) in this location, 115 TEs (**Figure 1**) were selected for the present study covering 62.84% of the totaltea-cultivated area (**Supplementary Table S1**). Altogether 7300 topsoil (0-15 cm) samples were collected following the standard protocol (Karak et al., 2015) and references therein. The detailed information on the sampling site is presented in **Figure 1**. In brief, soil samples were collected using wooden agar and subjected to air-drying. The dried soil samples were then grounded and passed through a 2-mm nylon sieve. The sieved samples (<2 mm) were stored in a polyethylene zip bag and stored in an air-ventilated room before analysis. The soil samples were analyzed for pH, organic carbon (OC), available potassium using neutral normal NH_4_OAc (AK), and available sulphur (AS) using 500 mg L^-1^ P (KH_2_PO_4_) solution. The pH of the soils was determined by using soil water suspension in the ratio of 1:2.5 using the protocol described by Jackson (1973). The OC content of the soils was estimated as described by Nelson and Sommers (1982). The protocol described by Pratt (1965) and Ensminger (1954) was followed for the estimation of AK (as K_2_O) and AS, respectively.

### Interpretation of the data sets and statistical analysis

#### Categories of soil parameters

The chemical properties of the soil reaction (pH), oxidizable organic carbon, and nutrient status (K and S) are classified based on the rating chart like low, medium, and high for tea cultivation. In this study, an attempt was made to create micro-level categories of each parameter (**Supplementary Table S2**) to understand the exact status of the parameters for better soil management and appropriate application of inputs.

#### Nutrient index

Analyzed data of soil pH, OC, AK, and AS were used to evaluate the fertility status of soils in the sub-districts of Dooars by calculating the nutrient index. The nutrient index was calculated following the equation proposed by Ramamoorthy and Bajaj (1969):


(1) $$Nutrient\;index=\frac{\left(L\times 1)+(M\times 2)+(H\times 3\right)}{Total\;number\;of\;soil\;samples}$$

where, L= No. of soil samples of low status; M= No. of soil samples of medium status and H= No. of soil samples of high status

Nutrient index based on the soil test values were categorized into low, medium, and high (**Supplementary Table S3**).

#### Statistical analysis

First, the pair wise association among the soil parameters [pH, organic carbon (OC), available potassium (AK), and available sulphur (AS)] in the different scenarios have been examined using Pearson’s correlation coefficient and their significance have been checked with the help of t-test. Pair wise correlations of the soil parameters against different sub-district, categories of soil pH, categories of soil organic carbon (OC), categories of soil available potassium (AK), and categories of soil available sulphur (AS) have been computed to understand the linear association in different viewpoints.

To check the significant difference among the different sub-districts concerning the level of soil parameters, one-way anova followed by the Duncan’s Multiple Range Test (DMRT) test (as a *post hoc* test) has been implemented. The DMRT test was carried out with a 5% level of significance.

Logistic regression was employed to model the derived nutrient index to quantify the effect of soil parameters (pH, organic carbon, available potassium, and available sulphur) on the nutrient index. When the study variable is ordinal in nature, logistic regression, introduced by Cox (1958) and Walker and Duncan (1967), is used over the traditional regression model, due to its loose assumption for homoscedasticity, normality, and linearity. In our case, the variable of interest is nutrient class i.e., low, medium, and high. So, multinomial logistic regression has been applied. The statistical equation of multinomial logistic regression can be represented by equation 2


(2) Y_i=β+β_1 x_1+···+β_p x_p+ϵ_i

Where, Y_i is the response variable; x_1,x_2,…,x_p are the regressors variables, β_1,β_2,…β_p are the respective coefficient and ϵ_i is error term. Let π_i denote the probability that Y_i = 1 given X = (1,x_1,x_2…,x_p). The logistic function is used to examine the relation between the probability π_i and X. Logistic function is an S-shaped curve and is represented in equation 3.


(3) $$\begin{array}{c} \pi \_i=1/(1+e^{\wedge }(-(\beta +\beta \_1\;x\_1+\cdot \cdot \cdot \\ \;\;+\beta \_p\;x\_p))\;);-\infty \\ \;\;<(\beta +\beta \_1\;x\_1+\cdot \cdot \cdot +\beta \_p\;x\_p)<\infty \end{array}$$

In our case, Y_i has three categories; low, medium, and high. A number of independent variables, p, is four i.e., pH, OC, AK, and AS.

The general graph, ANOVA, Box plot, correlation, density, histogram, scatter, and logistic regression. This statistical analysis was performed using the MS EXCEL, IBM SPSS Statistics 26 statistical package (SPSS Inc., USA), and R Programming version 4.2.0 supported by the R Foundation for Statistical Computing (Indianapolis, Indiana, USA).

## Results

### Assessment of soil fertility status

#### Status of soil pH in different sub-districts in Dooars tea growing region

**Figure 2:**
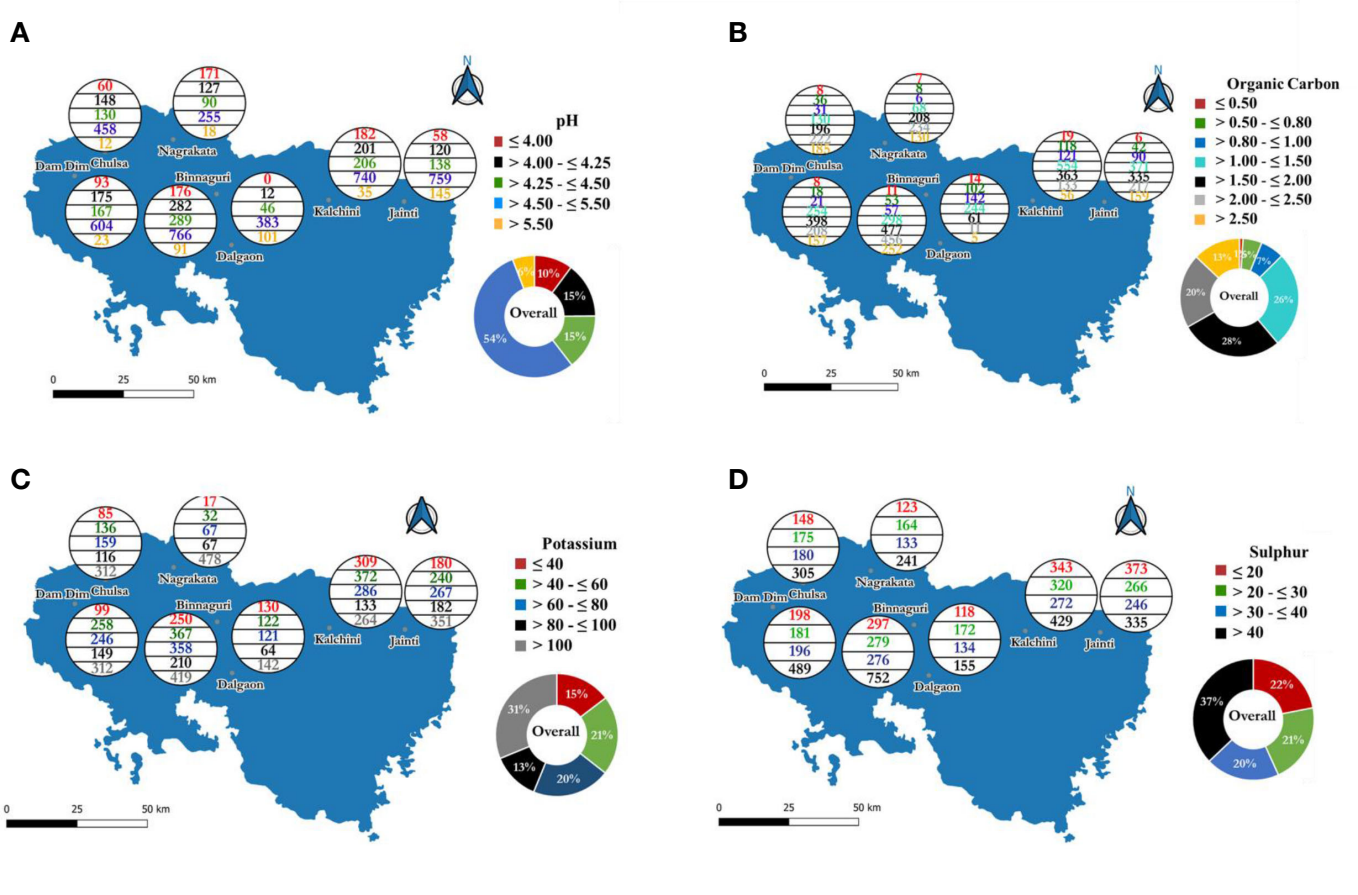
Distribution pattern of7300 analyzed soil samples collected from seven sub-districts in the tea growing region of Dooars, West Bengal, India based on different ranges of reported soil parameters:**(A)** pH, **(B)** organic carbon, **(C)** available potassium and **(D)** available sulphur.

Overall soil pH ranged between 3.00 and 8.44 (**Table 1**). The pH of soil samples for Dam Dim, Chulsa, Nagrakata, Binnaguri, Dalgaon, Kalchini, and Jaintiranged from 3.30 to 6.82, 3.48 to 6.73, 3.33 to 8.05, 3.00 to 8.44, 3.58 to 7.97, 3.30 to 7.53 and 3.50 to 8.42, respectively. The mean pH values of respective sub-districts were within the range between 4.38 and 5.03. The categorized soil pH status in different sub-district is depicted in **Figure 2A** (data against **Figure 2A** has been tabulated in **Supplementary Table S4**). It was observed that around 25.87% of the analyzed soils collected from the Nagrakata sub-district showed very low soil pH (≤ 4.00) followed by Kalchini (13.34%), Binnaguri (10.97%), Dam Dim (8.74%), Chulsa (7.43%), Jainti (4.75%) and Dalgaon (2.07%). It was also evident that 10.30% of the gardens were found under the very low soil pH and 14.93% of soils were found in the moderately low soil pH (>4.00-≤4.25) category. The per cent contribution of moderately low soil pH (>4.00-≤4.25) in sub-districts followed the trend as Nagrakata (19.21%) >Chulsa (18.32%) > Binnaguri (17.58%), Dam Dim (16.45%) > Kalchini (14.74%) > Jainti (9.84%) > Dalgaon (6.39%). Around 18.02% of soil samples collected from the Binnaguri sub-district showed low soil pH (>4.25-≤4.50) followed by Chulsa (16.09%) sub-district. The per cent of optimal soil pH (>4.50-≤5.50) among the seven sub-districts ranged from 38.58 (Nagrakata) to 66.15 (Dalgaon) which corresponds to 54.32% of total analyzed soil samples. Soil pH (>5.50) was found (17.44%) in the soil samples collected from the Dalgaon sub-district. Therefore, around 45.68% of total analyzed soil samples were found to have either lower or higher soil pH than soil pH suitable for tea cultivation.

**Table 1:** Descriptive statistics of the measured soil properties in the Dooars tea growing region of West Bengal, India covering seven sub-districts.

	Name of the sub-districts													
Parameters^*^	Binnaguri		Chulsa		Dalgaon		Dam Dim		Jainti		Kalchini		Nagrakata	
	Range	Mean ± SE	Range	Mean ± SE	Range	Mean ± SE	Range	Mean ± SE	Range	Mean ± SE	Range	Mean ± SE	Range	Mean ± SE
pH	3.00-8.44	4.57^c^ ± 0.60	3.48-6.73	4.54^cd^ ± 0.41	3.58-7.97	5.03^a^ ± 0.72	3.30-6.82	4.54^cd^ ± 0.43	3.50-8.42	4.88^b^ ± 0.85	3.30-7.53	4.51^d^ ± 0.47	3.33-8.05	4.38^e^ ± 0.64
OC (%)	0.31-5.86	1.94^c^ ± 0.71	0.39-6.00	2.03^b^ ± 0.82	0.31-4.52	1.11^f^ ± 0.41	0.28-5.84	1.90^c^ ± 0.71	0.31-4.40	1.73^d^ ± 0.64	0.31-4.70	1.45^e^ ± 0.54	0.29-4.75	2.10^a^ ± 0.64
AK (mg kg^-1^)	19-501	87.45^c^ ± 61.38	16-375	100.38^b^ ± 60.49	15-500	84.87^c^ ± 66.22	22-390	88.07^c^ ± 48.63	16-417	86.86^c^ ± 52.77	18-475	73.86^d^ ± 48.82	23-495	159.28^a^ ± 90.18
AS (mg kg^-1^)	5-165	43.47^a^ ± 24.74	5-150	40.71^b^ ± 23.45	5-168	37.66^c^ ± 25.08	5-145	43.44^a^ ± 24.63	5-163	33.69^d^ ± 21.60	5-155	37.48^c^ ± 23.60	5-171	41.68^ab^ ± 25.90

^*^pH (Soil: Water::1.0:2.50); OC, Organic carbon; AK, available K as K_2_O; AS, available S. (Mean values represent the mean of three replications ± SE; the same symbol in a column indicates non-significant difference among the variables whereas different symbol indicates significant differences among the variables at 5% level of significance).

#### Status of soil organic carbon (OC) content in different sub-districts in Dooars

Soil organic carbon plays a pivotal role to maintain soil health. The content of soil organic carbon (%) ranged from 0.28 to 5.84, 0.39 to 6.0, 0.29 to 4.75, 0.31 to 5.86, 0.31 to 4.52, 0.31 to 4.40, and 0.31 to 4.70 in the soils of Dam Dim, Chulsa, Nagrakata, Binnaguri, Dalgaon, Kalchini and Jainti sub-districts, respectively (**Table 1**). Based on soil organic carbon data (in %), the analyzed soil samples were classified as very poor (≤0.50), poor (>0.50-≤0.80), satisfactory (>0.80-≤1.00), moderately good (>1.00-≤1.50), satisfactory good (>1.50-≤2.00), good (>2.00-≤2.50), and very good (>2.50). The number of soil samples in each sub-district fall into different categories of soil organic carbon has been depicted in **Figure 2B** (data against **Figure 2B** has been tabulated in **Supplementary Table S5**). Around 1% of the total analyzed samples were found to have<0.50% (very poor) organic carbon content. Among the analyzed soil samples, it was observed that a maximum of 2.42% of soil samples of the Dalgaon sub-district is having very poor organic carbon content. However, only 0.49% of total soil samples collected from Jainti sub-district soils showed very poor organic carbon. The per cent of analyzed soil samples having poor organic carbon content (>0.50-≤0.80% OC) ranged between 1.21 (Nagrakata) and 17.62 (Dalgaon). A satisfactory organic carbon content (>0.80-≤1.00%) was found in 6.41% of total soil samples in the Dooars tea growing areas. Moreover, the analyzed data also showed that 24.53, 8.87, and 7.38% of soil samples of Dalgaon, Kalchini, and Jainti sub-districts were found under satisfactory organic carbon levels. Further, it was observed that 26.29% of soil samples of the entire Dooars region soils were found under the moderately good category (>1.00-≤1.50%). This category of soil was found more in the Dalgoan sub-district (42.14%) followed by Kalchini (40.62%) and Jainti (30.41%) sub-districts. Whereas, satisfactory good category (>1.50 - ≤2.00%) soils were found more in Dam Dim sub-district (37.41%) followed by Nagrakata (31.47%) and Binnaguri (29.74%) sub-districts. On average, 27.92% of analyzed soil samples were found satisfactory good category of organic carbon content. The analyzed data indicated that the higher percentage of soils under the good category (>2.00-≤2.50%) were found in Nagrakata (35.40%) followed by Binnaguri (28.43%) and Chulsa (27.48%) sub-districts. On the other hand, 12.93% of soil in the Dooars region was found in the very good category (>2.50%) of soil organic carbon content. It was observed that Chulsa (22.90%) followed by Nagrakata (19.67%), Binnaguri (15.71%), Dam Dim (14.76%), and Jainti (13.03%) sub-districts were found to have very good organic carbon content.

#### Status of soil available potassium (AK) content in different sub-districts in Dooars

The descriptive statistics of soil-available potassium (AK) have been tabulated in **Table 1**. The content of AK in soil varied from 22 to 390, 16 to 375, 23 to 495, 19 to 501, 15 to 500, 16 to 417, and 18 to 475 mg kg^-1^ with mean values of 88.1 ± 48.6, 100.4 ± 60.5, 159.3 ± 90.2, 87 ± 66.2, 84.9 ± 66.2, 73.9 ± 48.82 and 86.9 ± 52.8 mg kg^-1^ in Dam Dim, Chulsa, Nagrakata, Binnaguri, Dalgaon, Kalchini and Jainti sub-districts, respectively. The content of very low (≤40 mg kg^-1^) and low-level (>40-≤60 mg kg^-1^) AK for tea in the soils of Dooars were found as 14.66% and 20.92%, respectively (**Figure 2C**, data against **Figure 2C** has been tabulated in **Supplementary Table S6**). The analyzed data also indicated that a higher percentage of very low levels of AK content was found in the tea-growing soils of Kalchini (22.65%) followed by Dalgaon (22.45%), Binnaguri (15.59%), and Jainti (14.75%) sub-districts. Whereas, a higher percentage of the low content of AK (>40 - ≤60 mg kg^-1^) was found in the soils of the Kalchini sub-district (27.27%) followed by Dam Dim (24.25%), Binnaguri (22.88%), and Dalgaon (21.07%) sub-districts. Around 20.60% low medium (>60-≤80 mg kg^-1^) and 12.62% high medium (>80-≤100 mg kg^-1^) categories of AK content of total analyzed soil samples were found in the soils of Dooars. The low-medium category AK content ranged between 10.14% (Nagrakata) and 23.12% (Dam Dim). The high medium AK (>80-≤100 mg kg^-1^) category was recorded more in the soils of Jainti sub-districts (14.92%) followed by Chulsa (14.36%) and Dam Dim (14.00%) sub-districts. Around 31.21% of total analyzed soils were under high AK (>100 mg kg^-1^) content. It was observed that 72.31% of soils in the Nagrakata sub-district have high levels of AK followed by Chulsa (38.61%) and Dam Dim (29.32%) sub-districts.

#### Status of soil available sulphur (AS) content in different sub-districts in Dooars

Soil can be classified into four classes, viz. very low (≤20 mg kg^-1^), low (>20-≤30 mg kg^-1^), medium (>30-≤40 mg kg^-1^), and high (>40 mg kg^-1^) based on AS content (**Supplementary Table S7**). The mean values of available sulphur (AS) content ranged from 33.69 to 43.47 mg kg^-1^ in the soils of seven sub-districts of Dooars (**Table 1**). The highest mean value of AS was observed in the soils of the Binnaguri sub-district. The 21.92, 21.33, 19.68, and 37.07% of analyzed soils were found under very low, low, optimum, and high categories, respectively (**Figure 2D**, data against **Figure 2D** has been tabulated in **Supplementary Table S7**). It was observed that a higher percentage of soil samples under low (29.71%) and medium (23.14%) levels of AS content was found in the Dalgaon sub-district (**Figure 2D**). However, the lowest content of low and medium levels of AS was found in Dam Dim (17.01%) and Binnaguri (17.21%). It was observed that the highest percentage of the high-category AS content was found in Binnaguri (46.88%). In contrast, a lower percentage was seen in Dalgaon sub-district soils (26.77%) among the analyzed soil samples.

### Assessment of soil fertility based on the nutrient index

Overall nutrient indexes concerning pH, OC, AK, and AS are tabulated in **Table 2**. Nutrient index analysis for the tea growing areas of Dooars indicated that the percent of soil was higher and lower under the low soil pH category in Nagrakata sub-district (58.70%) and Dalgaon (16.41%) sub-districts, respectively (**Table 2**). A higher per cent of soil samples under medium and high pH categories in the Dalgaon sub-district was found at 66.2% and 17.4%, respectively. The low nutrient index of pH was found in Binnaguri, Chulsa, Dam Dim, Jainti, and Nagrakata sub-districts. Soil collected from Dalgaon and Kalchini sub-districts was found in the medium range of nutrient index concerning soil pH. In the case of soil OC, the per cent of soil samples was higher under low and high soil OC content categories in Dalgaon (44.56%) and Nagrakata (86.54%) sub-districts, respectively. However, most of the sub-districts indicated a high (2.47 to 2.83) nutrient index of soil OC. The medium index concerning OC was found only in Dalgaon (1.69), and Kalchini (2.22) sub-districts. In the Nagrakata sub-district, 72.31% of soil samples were found under the high category of AK content with a 2.65 nutrient index. However, in the rest of the sub-districts, the nutrient index for AK varied from 1.69 to 2.11, indicating a medium nutrient index. In the case of AS, the medium nutrient index was recorded in all the sub-districts with a range varied between 1.97 and 2.28. Nevertheless, a higher percentage of soil samples (46.88%) with AS content was found in the Binnaguri sub-district.

**Table 2:** Frequency of soil samples falling in the indices categories, percent (%) of soil samples under each category, and Nutrient Index value as well as rating in the Dooars tea growing region of West Bengal, India covering seven sub-districts based on soil pH, organic carbon (OC), available K as K_2_O (AK) and available S (AS) (total number of soil samples: 7300; the total number of tea estates: 115 and number of replications for each sample:3).

Sub-districts	Number of samples falling in the index categories			Percent (%) of soil samples under each category			Nutrient Index^*^	
Low	Medium	High	Low	Medium	High	Value	Rating
pH								
Binnaguri	747	766	91	46.57	47.76	5.67	1.59	Low
Chulsa	338	458	12	41.83	56.68	1.49	1.60	Low
Dalgaon	95	383	101	16.41	66.15	17.44	2.01	Medium
Dam Dim	435	604	25	40.88	56.77	2.35	1.61	Low
Jainti	316	759	145	25.90	62.21	11.89	1.86	Low
Kalchini	589	740	35	43.18	54.25	2.57	1.59	Medium
Nagrakata	388	255	18	58.70	38.58	2.72	1.44	Low
Total	2908	3965	427	39.84	54.32	5.85	1.66	Medium
Organic carbon (OC)								
Binnaguri	121	298	1185	7.54	18.58	73.88	2.66	High
Chulsa	75	130	603	9.28	16.09	74.63	2.65	High
Dalgaon	258	244	77	44.56	42.14	13.3	1.69	Medium
Dam Dim	47	254	763	4.42	23.87	71.71	2.67	High
Jainti	138	371	711	11.31	30.41	58.28	2.47	High
Kalchini	258	554	552	18.91	40.62	40.47	2.22	Medium
Nagrakata	21	68	572	3.18	10.29	86.54	2.83	High
Total	918	1919	4463	12.58	26.29	61.14	2.49	High
Available K as K_2_O (AK)								
Binnaguri	617	568	419	38.47	35.41	26.12	1.88	Medium
Chulsa	221	275	312	27.35	34.03	38.61	2.11	Medium
Dalgaon	252	185	142	43.52	31.95	24.53	1.81	Medium
Dam Dim	357	395	312	33.55	37.12	29.32	1.96	Medium
Jainti	420	449	351	34.43	36.80	28.77	1.94	Medium
Kalchini	681	419	264	49.93	30.72	19.35	1.69	Medium
Nagrakata	49	134	478	7.41	20.27	72.31	2.65	High
Total	2597	2425	2278	35.58	33.22	31.21	1.96	Medium
Available S (AS)								
Binnaguri	297	555	752	18.52	34.60	46.88	2.28	Medium
Chulsa	148	355	305	18.32	43.94	37.75	2.19	Medium
Dalgaon	118	306	155	20.38	52.85	26.77	2.06	Medium
Dam Dim	198	377	489	18.61	35.43	45.96	2.27	Medium
Jainti	373	512	335	30.57	41.97	27.46	1.97	Medium
Kalchini	343	592	429	25.15	43.40	31.45	2.06	Medium
Nagrakata	123	297	241	18.61	44.93	36.46	2.18	Medium
Total	1600	2994	2706	21.92	41.01	37.07	2.15	Medium

^*^For Nutrient Index rating please see **Table S3**.

### Assessment of soil character by correlation analysis

**Figure 3:**
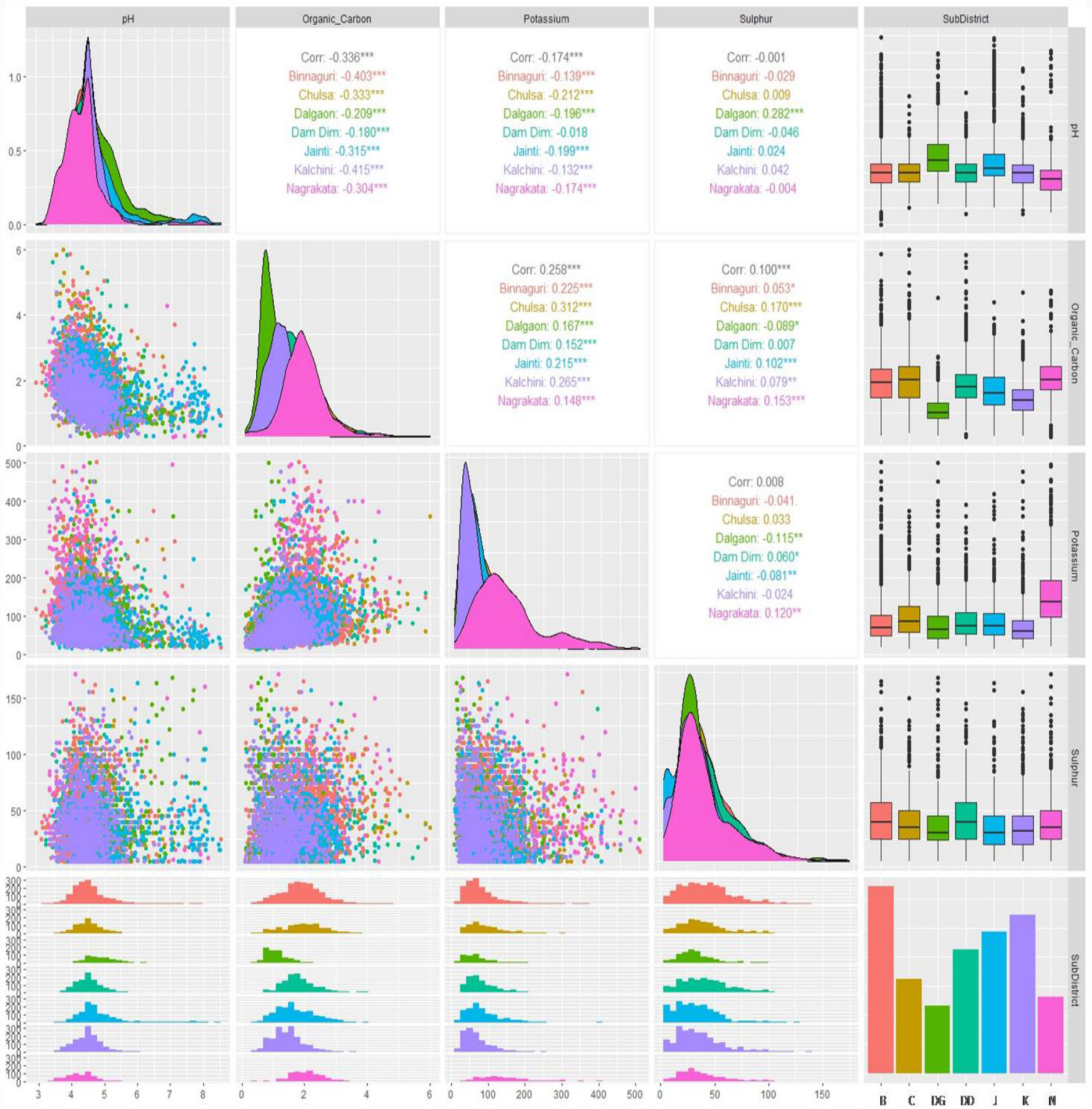
Sub-district wise bivariate correlations of reported soil parameters (pH, organic carbon, available potassium, and available sulphur) of 7300 analyzed soil samples represented by box plot, density, scatter, histogram, and bar diagram (in bar diagram B, Binnaguri; C, Chulsa; DG, Dalgaon; J, Jainti; K, Kalchini and N, Nagrakata; here ‘Organic_Carbon’ is ‘OC’, ‘Potassium’ is ‘available potassium (K) as K_2_O’ abbreviated as ‘AK’ and Sulphur is ‘available sulphur’ abbreviated as ‘AS’ in the manuscript; significance codes: “***” at 0.001%, “**” at 0.01% and “*” at 0.05% levels).

Sub-district-wise bivariate correlations of reported soil parameters [pH, organic carbon (OC), available potassium (AK), and available sulphur (AS)] of 7300 analyzed soil samples have been presented in **Figure 3**. The results showed that the soil pH was significantly and negatively correlated (r=0.336) with soil OC. The results revealed that a highly negative and significant correlation was found between soil pH and OC in Kalchini sub-district soils (r=-0.415) followed by Binnaguri (r=-0.403), Chulsa (r=-0.333), Jainti (r=-0.315), Nagrakata (r=-0.304), Dalgaon (r=-0.208) and Dam Dim (r=-0.180). The soil pH was significantly and negatively correlated (r=-0.174) with soil AK. Among the sub-districts, only soil collected from the Dam Dim sub-district showed a non-significant and negative (r=-0.018) correlation. However, soils from other sub-districts followed a significant and negative correlation when soil pH and AK were considered. In the case of soil pH with AS, only Dalgaon sub-district soils were significantly and positively correlated (r=0.282) but soils from the other six sub-districts were found to be non-significantly correlated (r=-0.001). The soil OC was significantly and positively correlated with soil AK in all analyzed soil samples (r=0.258). Among the sub-districts, the soils collected from Chulsa sub-district showed maximum strong correlation (r=0.312) followed by Kalchini (r=0.265), Binnaguri (r=0.225), Jainti (r=0.215), Dalgoan (r=0.167), Dam Dim (r=0.152) and Nagrakata (r=0.148). The soil OC was significantly and positively correlated with AS for Chulsa (r=0.170), Nagrakata (r=0.153), Jainti (r=0.102), Kalchini (r=0.079) and Binnaguri (r=0.053) sub-districts. A significant negative correlation was found only under Dalgaon sub-district soils (r=-0.089) but there was no significant correlation found in the case of Dam Dim when OC and AS were considered. The overall correlation between AK and AS was found to be non-significant and positive (r=0.008). However, a significant negative correlation between AK and AS was found in the soils of Dalgaon (r=-0.116) and Jainti (r=-0.081) sub-districts. A significant positive correlation was observed in the soils of Nagrakata (r=0.120) and Dam Dim sub-districts (r=0.060).

**Figure 4:**
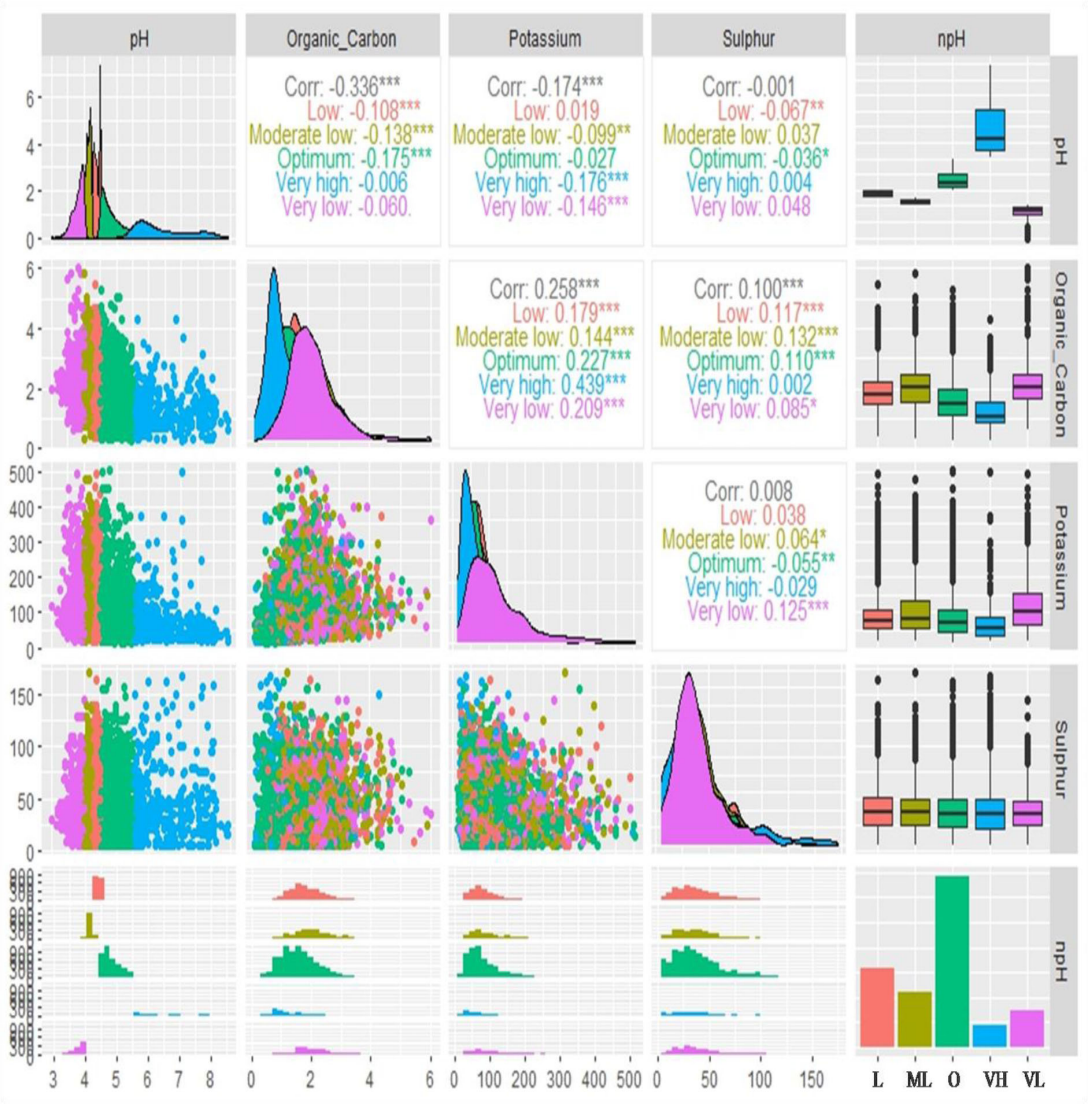
Bivariate correlations of reported soil parameters (pH, organic carbon, available potassium, and available sulphur) of 7300 analyzed soil samples against different categories of soil pH represented by box plot, density, scatter, histogram, and bar diagram (in bar diagram L, Low; M, Moderately Low; O, Optimum; VH, Very high and VL, Very low; here ‘Organic_Carbon’ is ‘OC’, Potassium is ‘available potassium (K) as K_2_O’ abbreviated as ‘AK’ in the manuscript and Sulphur is ‘available sulphur’ abbreviated as ‘AS’ in the manuscript; Significance codes: “***” at 0.001%, “**” at 0.01% and “*” at 0.05% levels).

The correlation analysis among pH, OC, AK, and AS under different categories of soil pH has been presented in **Figure 4**. It was observed that optimum soil pH was highly significant and negatively correlated with soil OC (r=-0.175). The very high soil pH and very low soil pH were found to be highly significant and negatively correlated with AK. On the other hand, the low soil pH was found to be significantly and negatively correlated (r=-0.067) with AS. Soils under very high pH category, the OC and AK were highly significant positively correlated (r=0.439) followed by optimum (r=0.227), low (r=0.179), moderately low (r=0.144) and very low (r=0.029) soil pH. When examined, it was found that under moderately low pH, a strong positive correlation was found between OC and AS (r=0.132) followed by under low (r= 0.117), optimum (r=0.110), and very low (r=0.085) soil pH levels. However, under very low pH levels, the AK and AS were positively correlated to each other but a negative correlation was found with very high and optimum soil pH levels.

**Figure 5:**
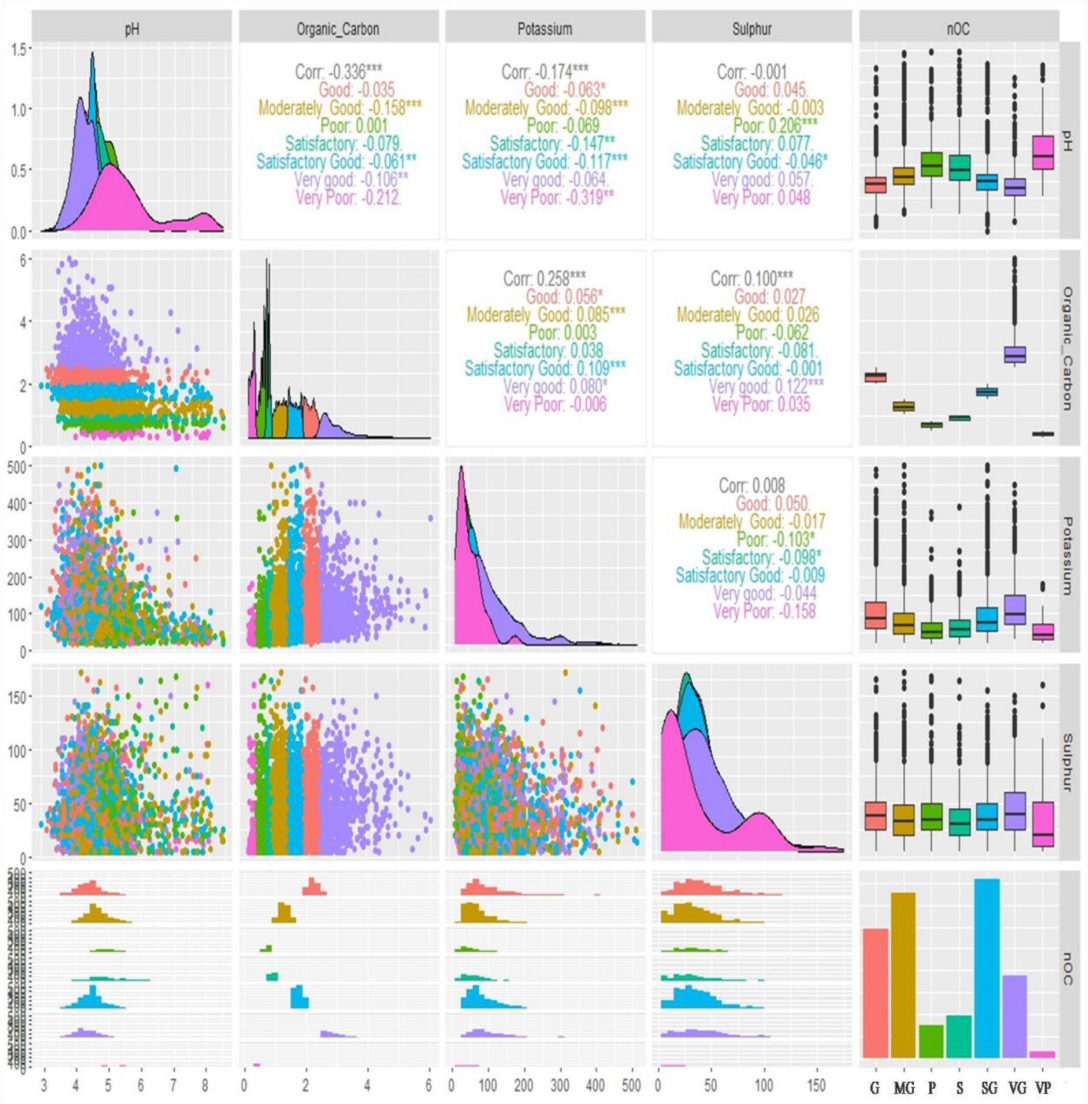
Bivariate correlations of reported soil parameters (pH, organic carbon, available potassium, and available sulphur) of 7300 analyzed soil samples against different categories of soil organic carbon (OC) represented by box plot, density, scatter, histogram and bar diagram (in bar diagram G, Good; MG, Moderately Good; P, Poor; S, Satisfactory; SG, Satisfactory Good; VG, Very Good and VL, Very poor; here ‘Organic_Carbon’ is ‘OC’, Potassium is ‘available potassium (K) as K_2_O’ abbreviated as ‘AK’ in the manuscript and Sulphur is ‘available sulphur’ abbreviated as ‘AS’ in the manuscript; Significance codes: “***” at 0.001%, “**” at 0.01% and “*” at 0.05% levels).

The correlation analysis among pH, OC, AK, and AS under different categories of soil OC has been presented in **Figure 5**. It was found that the moderately good OC level of soil was highly significant and negatively correlated with soil pH (r=-0.158) followed by very good OC (r=-0.106) and satisfactory good OC (r=-0.061). Other categories of soil OC were found to be non-significant and negatively correlated with soil pH except poor OC which showed a non-significant and positive correlation. However, under the very poor soil OC category, a highly negative correlation was found between soil pH and AK (r=-0.319) but a positive and significant correlation (r=0.098) was found between soil pH and AK under the moderately good OC category. Notwithstanding, no strong relationship was found between soil pH and AS irrespective of categories of soil OC. A positive and significant correlation (r=0.206) was found between soil pH and AS under poor OC levels. In the satisfactory good category soil OC, a positive and significant correlation was found between OC and AK (r=0.109) followed by moderately good OC (r=0.085), very good OC (r=0.080), and good OC (r=0.056) categories. The OC was significantly and positively correlated with AS (r=0.100), and very good category soil OC was only significantly and positively correlated (r=0.122) with AS. In general, the AS and AK were non-significantly and positively correlated (r=0.008). A non-significant positive correlation was found (r=0.050) only under the good category soil OC. A negative correlation was found between AK and AS in the rest of the categories of soil OC.

**Figure 6:**
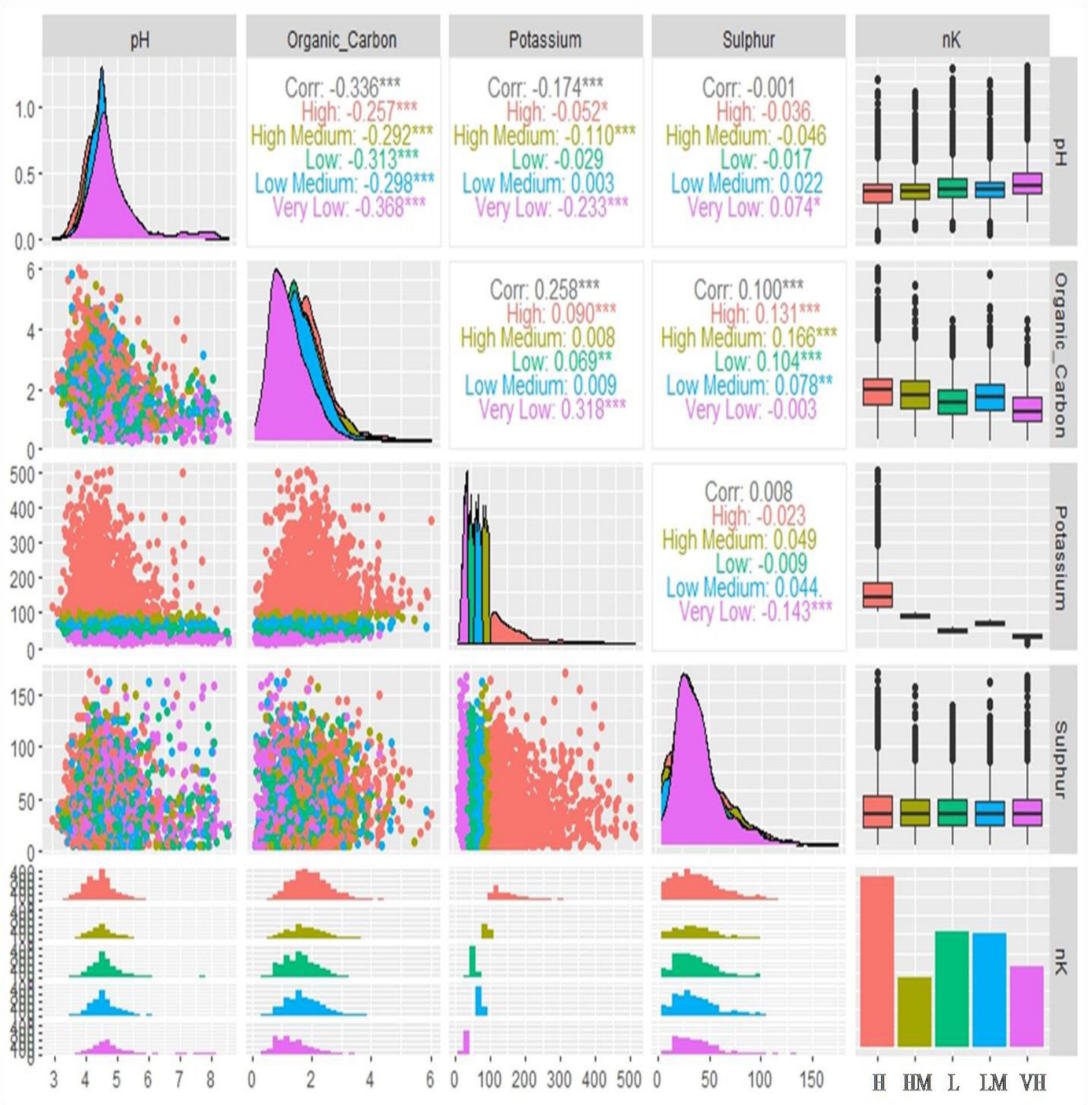
Bivariate correlations of reported soil parameters (pH, organic carbon, available potassium, and available sulphur) of 7300 analyzed soil samples against different categories of soil available potassium (AK) represented by box plot, density, scatter, histogram and bar diagram (in bar diagram H, High; HM, High Medium; L, Low; LM, Low medium and VL, Very low; here ‘Organic_Carbon’ is ‘OC’, Potassium is ‘available potassium (K) as K_2_O’ abbreviated as ‘AK’ in the manuscript and Sulphur is ‘available sulphur’ abbreviated as ‘AS’ in the manuscript; Significance codes: “***” at 0.001%, “**” at 0.01% and “*” at 0.05% levels).

The correlation analysis among pH, OC, AK, and AS under different categories of soil AK has been depicted in **Figure 6**. The relation between soil pH and OC was found to be highly significant and negative under of very low soils AK category (r=-0.368) followed by low AK (r=-0.313), low medium AK (r=-0.298), high medium AK (r=-0.292), and high AK (r=-0.257) categories. On the other hand, soil pH correlated highly significantly and negatively under the very low category of soil AK (r=-0.233) followed by the high medium AK category (r=-0.110). The soil pH and AS were found to exhibit a non-significant negative correlation (r=-0.001). Under very low soil AK content, a significant and positive correlation was found between soil pH and AS (r=0.074). On the other hand, very low soil AK content was also found to be highly, significantly, and positively correlated with OC (r=0.318) category followed by high (r=0.090) and low (r=0.069) categories soil AK. Whereas, a highly significant and positive correlation was found between soil OC and AS under high medium AK content (r=0.166) followed by high (r=0.131) and low (r=0.104) levels of AK. A significant and negative correlation between AK and AS (r=-0.143) was found under the very low AK category.

**Figure 7:**
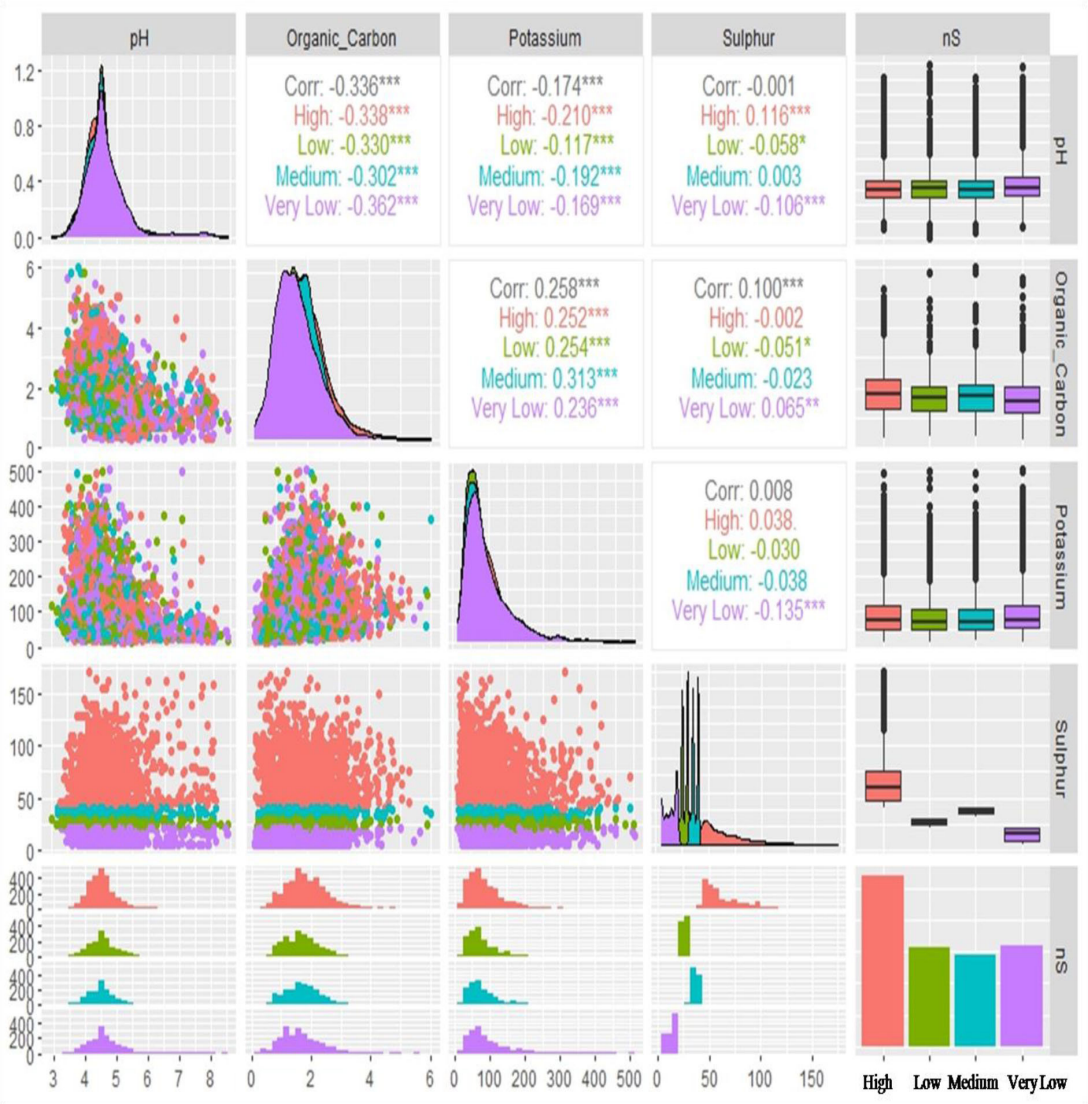
Bivariate correlations of reported soil parameters (pH, organic carbon, available potassium and available sulphur) of 7300 analyzed soil samples against different categories of soil available sulphur (AS) represented by box plot, density, scatter, histogram and bar diagram: here ‘Organic_Carbon’ is ‘OC’, Potassium is ‘available potassium (K) as K_2_O’ abbreviated as ‘AK’ in the manuscript and Sulphur is ‘available sulphur’ abbreviated as ‘AS’ in the manuscript; Significance codes: “***” at 0.001%, “**” at 0.01% and “*” at 0.05% levels).

Lastly, **Figure 7** represents the correlation analysis among pH, OC, AK, and AS under different categories of soil AS. Under very low content of AS, the soil pH and OC were correlated highly and negatively (r=-0.362) followed by high (r=-0.338), low (r=-0.330), and medium (r=-0.302) levels of AS categories. A significant and negative correlation was observed between soil pH and soil AK under all levels of soil AS. Under the high level of AS category, a positive significant correlation was found between pH and AS. Overall a significant positive correlation was found between OC and AK under different categories of soil AS. A positive and significant correlation was found between OC and AS (r=0.065; p< 0.05) under very low levels of soil AS and a negative correlation was found in the resting level of AS categories of soil. Under the very low level of soil AS a significant negative (r=-0.135; p< 0.001) correlation was observed between AK and AS.

## Discussion

### Soil pH

It has been observed that around 40% of the soil samples collected from 115 TEs spread over seven sub-districts of Dooars regions were found to be having pH below the optimum level of soil pH (4.50 to 5.50) essential for tea cultivation (Karak et al., 2015). Therefore, soils having pH<4.50 need proper corrective measures to get optimum tea production. Several reported results concluded that the causes of high acidity (pH<4.50) in tea-growing soils could be due to acidic parent materials, high precipitation, presence of soil exchangeable aluminium and manganese, leaching of basic cations, and indiscriminate uses of acid-forming fertilizers, viz. high rate of N fertilization (Barooah and Gohain, 1999; Karak et al., 2011; Karak et al., 2015; Zhao et al., 2018b; Yan et al., 2020). According to Ruan et al. (2004), the high pH decline in tea-growing soil might be related to tea plant physiology as well as the release of organic acids, carbonic acid, and polyphenols from tea root to its rhizospheric zone. Furthermore, Wang et al. (2010) reported that the decomposition of Al-containing tea litter in soil enhances soil acidification in tea plantations. Soil acidification can adversely affect nutrient availability in plants (Kemmitt et al., 2005) when the soil pH drops to less than 4.0, which subsequently affects both the quality and quantity of tea production (Fung et al., 2008; Yan et al., 2018; Yan et al., 2020). A recent study at tea-growing regions of Anxi, Songyang, and Wuyi in China extending between latitudes 18-36^0^ N and longitudes 94-121^0^ E reported significant soil acidification between the 1980s and 2000s (Yan et al., 2020). The present report is also consistent with Zhu et al. (2018) where the authors highlighted the serious threat of soil acidification intea-growing regions resulting in low nitrogen use efficiency by tea plants. Furthermore, Kavitha et al. (2015) concluded that a significant deviation of soil pH from the required range could cause difficulties in the uptake of nutrients owing to changes in their chemical nature. In addition to nutrient unavailability, chemical decay of soil occurs when the soil pH goes below 4.5 and produces soluble salicylic acid and hydrated oxides of iron and alumina which also act as cementing materials for the formation of hard pans (Karak et al., 2015).

Where soils are low in soil pH (pH<4.50) can be amended by applying dolomite @ 2 Mg ha^-1^ once in a pruning cycle in the year of light pruning (Karak et al., 2015). The best result of liming is achieved by incorporating the dolomite uniformly at least 15 cm depth into the soil. Soils high in pH are recommended to apply iron pyrite or sulphur or aluminium sulphate @ 1-2 t ha^-1^ to reduce/decrease the soil pH level provided the drainage system is in order. An overdose of sulphur-containing fertilizers beyond the recommendation must be avoided which has a corrosive impact on the plant (Karak et al., 2015).

### Soil organic carbon (OC) status

Determining the factors affecting organic carbon (OC) storage in the soil of tea plantations is vital for reconnoitring approaches for increasing SOC sequestration, dropping soil carbon loss, and refining low-carbon planting in large-scale tea plantations (Di et al., 2020). Low-level soil OC is a significant challenge for sustainable soil health. The overall data of the study indicated that about 94% of soils of the Dooars region were between the satisfactory level and very good category of organic carbon. This may probably be attributed to the accumulation of organic carbon with a slow rate of decomposition (Singh et al., 2015). The data also suggested that the soil of Nagrakata, Dam Dim, Binnaguri, and Jainti sub-districts had a sufficient amount of organic carbon content compared to the rest of the sub-districts. The inappropriate cultivation practices, low input of organic manure and crop residues, and the rapid decomposition rate of organic matter might be responsible for inferior organic carbon content in the soils of some sub-districts of Dooars (Basak et al., 2021). Incorporation of different organic matter inadequate amounts, crop residue management, organic matter management within the garden, and a balanced fertilizer application will be helpful for the improvement of organic carbon for sustained productivity (Kanyenji et al., 2020). Maintaining a certain level of organic carbon is extremely important for soil fertility. Hence, monitoring the organic carbon level in the soil at the end of every pruning cycle is essential based on which necessary amendments and agricultural practices will be adopted.

Di et al. (2020) reported that iron oxide inhibits soil OC mineralization and protects soil OC from decomposition by soil microorganisms when the effects of the soil environment on soil OC were conducted in tea plantations located in Yunnan Province of south-western China. Furthermore, Zhang et al. (2019) corroborated the fact that different soil microbial community structures have different modes of soil OC decomposition, which affect soil OC sequestration capacity in soil. The nutrient-supplying capacity of organic matter to the tea plants depends on active or labile organic carbon pools. This labile carbon pool could be affected due to the soil age, continuous monoculture practices, and inadequate organic amendment in soil. The labile fractions of soil organic carbon can be used as soil quality indicators quickly changed with land-use management practice (Sharma et al., 2014; Li et al., 2016; Basak et al., 2021). Therefore, soil OC fractions are vital in sustaining soil quality under different management strategies (Bhattacharyya et al., 2011). Saha and Handique (2022) also reported that the seasonal variation in soil organic carbon differs from land cover to land cover in temperate North-eastern India.

To maintain the labile carbon in the soil, it is necessary to manage tea pruning litter properly and apply the minimum amount of organic manure in every pruning cycle despite the high organic carbon status in the soil (Borgohain et al., 2020). If litters of pruning and shade trees are retained *in situ*, soil organic carbon will increase, providing an energy source for soil microorganisms and leading to greater microbial biomass (Zhang et al., 2018). The role of these soil microorganisms in nutrient turnover organic matter, and transformations, including the synthesis of humic substances, is well recognised (Sanyal, 2001).

### Status of soil available potassium (AK)

Potassium (K) is a major macronutrient for tea’s metabolism processes which takes part in the quality and yield of tea plants. It also imparts resistance to tea plants in adverse situation (Li et al., 2021). The very low and low content of AK in these tea-growing areas under consideration might be due to the loose texture of the soil, inappropriate field management practices, and the low use of K fertilizers (Bagyalakshmi et al., 2017). On the other hand, the leaching condition brought in by high rainfall and strong acidity that does not permit K retention on the exchangeable soil complex might be the possible reason for the low K status of these soils. The imbalanced and continued use of superphosphate, even at a low dose of 45 kg ha^-1^y^-1^, can result in a 22 to 30% decrease in the total content of labile K and even at the low dose of 22.5 kg P_2_O_5_ per hectare over ten years or so resulted in about 3% decrease in the soil available K content, and about 20% decrease in the leaf concentration of potash (Dey, 1971). It has been observed that the significantly higher amount of AK in some soils of tea-growing regions of Dooars might be due to the well-practiced fertilizer management schedule (Hu et al., 2006). However, the location showing K deficiency needs special care in terms of K fertilizer management. In the present study, around 68.8% of the soils irrespective of the sub-districts were found to be deficient (≤100 mg kg^-1^) in soil AK, whereas, only 31.2% of the soils were having >100 mg kg^-1^ which is the required level of soil K for tea cultivation (Karak et al., 2015). Ravikumar and Somashekar (2013) found similar results (60.72 and 35.71% of soils under deficient and doubtful supply of soil AK) while evaluating nutrient index using OC, available phosphorus, and AK contents as a measure of soil fertility in Varahi River Basin, which is in the midst of Udupi district in the western part of Karnataka state, India. Therefore, to improve and build up K in the tea-growing soils, the K would be applied at the rate of 90 to 165 kg K_2_O ha^-1^ in four equal splits in a year based on the last pruning cycle yield to sustain the tea productivity. In the case of low, medium, and high medium soil AK, the K fertilizer may be applied in two splits in the ratio of 60:40 @ 70 to 140 kg K_2_O ha^-1^ according to the previous pruning cycle yield irrespective of soil types to maintain the soil solution K for plant uptake (Karak et al., 2015). The high content of AK recorded in Nagrakata and Chulsa sub-districts might be due to its parent material like oxisols (Histic Haplaquox) and the elevation of the location. However, in case of higher content of AK in the soil, the K fertilizer may be applied in two splits based on the previous pruning cycle yield level @ 50 -120 kg K_2_O ha^-1^ a year for maintaining the K level in soil and crop productivity (Karak et al., 2015).

### Soil available sulphur (AS) status

Sulphur (S) is one of the essential plant nutrients for the growth and production of tea and plays a significant role in tea quality as it is a constituent of amino acids such as cystine, cysteine, and methionine (Ananthacumaraswamy et al., 2003). To produce every tonne of made tea, the bushes have a biomass of about 5 tonnes, requiring about 10 kg S ha^-1^ (Bhat and Ranganathan, 1980; Barbora and Barua, 1988). However, every 3000 kg ha^−1^ crop of tea in Indian climatic conditions removes S@ 6 kg ha^−1^ annually as reported by Gohain and Dutta (1994).

In the present study, the AS content was found between 33.69 and 43.47 mg kg^-1^ in the soils of seven sub-districts of the Dooars region. Around 43.25, 19.68 and 37.07% of the total analyzed soil samples irrespective of the sub-districts were low, medium, and high in soil AS, respectively. Similar observations were also made by Patra et al. (2012) in which the soil samples of red and lateritic soils of West Bengal were found to be deficient in AS with an average of 45.2, 16.7, and 25.5% which were rated as low, medium, and high, respectively. The deficient and low levels of AS in soils of the tea growing areas of Dooars might be due to the application of less amount of sulphur-containing fertilizer in the soil and continuous removal of S by tea plants. In addition, low levels of AS in the soil might be responsible for the excessive use of urea instead of the ammonia sulphate (SA) fertilizer. Regular application of Sat 20-40 kg ha^-1^ with NPK fertilizers will be helpful to maintain the yield and quality of the tea (Phukan et al., 2009). The higher content of AS in some of the soils might be due to the high percentage of soil OC and fast S mineralization.

### Soil fertility based on nutrient index

The soil nutrient index value is a measure of the capacity of soil to supply nutrients to plants which also assesses the long-term impact of different crop-grown systems on changes in nutrient patterns (Amara et al., 2017; Singh et al., 2021). In this study, it was found that most of the examined soils are in low to medium pH index. Such kind of low pH index might be attributed to weathering of rock and minerals or the inherent properties of soil coupled with long-term tea monoculture cultivation practices. In this context, the adoption of appropriate soil management strategies would be helpful to improve the low and medium soil pH index of the soil. In the case of OC, the majority of soil samples of Dooars had a high index which might be attributed to the organic matter decomposition and continuous addition of organic matter to the soil in the form of leaf litter, pruning litter, green manuring, and some other organic manures like compost, vermicompost, biochar, etc. (Borgohain et al., 2020). This may also be due to the slow rate of soil organic matter decomposition in the region (Lal and Stewart, 2010). However, soils from some sub-districts of Dooars regions exhibited medium index and, therefore, need special attention for comprehensive soil organic matter management practices. The AK and AS index were found to be medium in Dooars soils. The study area received high precipitation during the monsoon season and due to such incidents, a huge amount of soluble K is leaching out from the soil. As a result, the nutrient use efficiency reduces drastically. In the Nagrakata sub-district, 72.31% of soil samples were found to record a high K index (2.65) which supports the findings of Sachan and Krishna (2022), reporting a high potassium index value (2.36) in Cassava Farm of Rewa Province of Fiji. To improve the nutrient index in respect of AK, the split application could be a promising one. The nutrient index value for soil AS was medium (1.97-2.27) in all the sub-districts. This result is in conformance with Dhotare et al. (2019) who also reported a higher percentage of soil samples (64%) under medium soil sulphur index (1.40) in the soils of Washim Road Farm of Dr. PDKV Akola, Maharashtra, India. This may be due to the application of less amount of sulphur-containing fertilizers in soil (Wagh et al., 2008). The low S index in tea-growing soil might be due to the slow rate of S mineralization in the acid tea soil, and consequently, the availability of S is quite low. In this regard, the application of appropriate S-containing fertilizers may be helpful to improve the S status in the region under study.

### Discussion on the correlation

This investigation found that the soil pH was significantly and negatively correlated with soil organic carbon (**Supplementary Figure S1**) in all sub-districts and all soils of Dooars with different levels of r values (**Figure 3**). Similar observations were reported by Zhou et al. (2020) when soil samples were collected from the Lancangjiang River Basin of Southwest China. The soil pH was significantly negatively correlated with soil AK (**Supplementary Figure S2**). Khadka et al. (2016) analyzed 172 soil samples from western Nepal and found a similar result. In general soil pH was negatively correlated with soil AS (**Supplementary Figure S3**). However, at the Dalgaon sub-district, soil pH was significantly positively correlated with soil AS (**Figure 3**). A significant and positive correlation between soil pH and AS was also found in western Nepal soil was also reported by Khadka et al. (2016). Moreover, a non-significant negative correlation was found between soil pH and AS in the soils of Dooars. In this context, Karak et al. (2015) also found that soil pH was negatively correlated with plant-available S in the Dhoedaam Tea Estate’s soil of Assam, India. The soil OC was significantly and positively correlated with AK in Dooars soils (**Supplementary Figure S4**). Similar results were also reported by Hossain et al. (2014) in the soils under Gamar forests in Bangladesh. In general, soil OC was positively correlated with soil AS (**Supplementary Figure S5**). The soil OC was significantly positively correlated with soil AS in Chulsa, Nagrakata, Kalchini, and Jainti sub-districts and entire Dooars soils. Bhat et al. (2017) also reported a similar type of observation in the correlation of available nutrients with physico-chemical properties and nutrient content of grape orchards of Kashmir. A similar result was also reported by Basumatari et al. (2010) on the soil of the Jorhat district of Assam. Overall data between AK and AS showed a positive correlation (**Supplementary Figure S6**). It was observed that AK and AS in soil collected from the Dam Dim sub-district showed a significant positive correlation at p< 0.05. Karak et al. (2015) also reported significant positive correlations between soil AK and AS in the Kamakhyabari tea estate soil of Assam.

Bivariate relationships between pH vs. OC, pH vs. AK, pH vs. AS, OC vs. AK, and OC vs. AS are presented in **Supplementary Figures S7A, E** and **Supplementary Tables S8**-**S12**. The analyzed data indicates that the higher soil OC content could reduce the soil pH. Alternatively, low pH benefits the accumulation of organic matter in the soil of tea plantations. However, the linear regression analysis indicated the behaviour of the soil nature concerning the relation between soil pH and OC of the studied area. It was indicated that when pH levels are increased (<4.00 to > 5.51), the percentage of soil samples steadily decreases soil OC content. This result might be due to the soil pH increases the solubility of soil organic matter by increasing the dissociation of acid functional groups (Evans et al., 2012). On the other hand, the solubility of organic matter is strongly influenced by the type of base and is notably more significant in the presence of monovalent cations than with multivalent ones (Curtin et al., 2016). However, the category-wise data indicated that when pH levels are increased (<4.00 to > 5.51), per cent of soil samples gradually increase under the very poor, poor, and satisfactory category of soil organic carbon levels. Moreover, the moderately good soil OC was increased up to an optimum pH level (pH-5.50), and then decreased the percentage of soil samples. However, at very low soil pH, a higher per cent of soil samples were found to have more than 1.51% organic carbon.

According to the linear correlation, the soil pH was significantly and negatively correlated with soil AK. The figure showed that the AK content was found in a major portion of soils within the soil pH (<4.00 to > 5.50) and gradually decreases with the increase of soil pH level. The category-wise wise analyzed data revealed that when pH levels are increased (<4.00 to > 5.51), per cent of soil samples repeatedly increased soil AK under the very low (≤ 40 mg kg^-1^) and low (>41 - ≤60 mg kg^-1^) category of soil. Whereas, in the case of low medium (>61 - ≤80 mg kg^-1^) and high medium (>81 - ≤100 mg kg^-1^), the per cent of soil samples was increased up to 4.49 soil pH afterward the soil samples per cent gradually decreased with the increase in soil pH. The analyzed data also revealed that at a very low soil pH level (<4.00), the high soil AK content (>100 mg kg^-1^) was found in more percentage of soils and gradually decreases with the increase of soil pH level. It was indicated that the soil pH influences the soil AK content. If the pH levels increase in the soil, the soil AK level also increases gradually. At the same time, the data also indicated that the influence of soil pH was more prominent on low to medium levels of AK compared to the high content of soil K. However, the availability of potassium depends upon the exchangeable potassium, soil texture, type of minerals, and soil moisture condition (Samuel and Ebenezer, 2014). Moreover, in general, the K was very less available at below 5.5 pH level. Because in strongly acid soils the tightly held H^+^ and hydroxy aluminum ions and potassium ions are closely associated with the colloidal surfaces. Therefore, the positive relationships between soil pH and AK were reported by previous researchers (Sharma et al., 2008; Athokpam et al., 2013), while negative relation was obtained by Singh and Mishra (2012) and Khadka et al. (2016). In this study, it was found that the potassium availability was much higher at low soil pH levels as compared to the higher soil pH level. The increase in potassium availability in the studied soil might be due to soil moisture content and soil temperature. At a very poor OC level, the highest per cent (50.68%) of soil samples was found very low (≤ 40 mg kg^-1^) content of AK. The per cent of soil samples gradually decreased with the increase in soil organic carbon content level. A more or less similar trend was also observed in the low content of AK. However, in the case of low medium (>60-≤80 mg kg^-1^) and high medium (>80-≤100 mg kg^-1^) soil AK content, the per cent of soil samples was increased up to good category organic carbon level. On the other hand, the available soil potassium content gradually increased with increasing the soil organic carbon level. According to the linear correlation, the soil pH was significantly and positively correlated with soil AK (r=0.258). These results indicate that organic matter significantly promotes the rate of potassium adsorption and desorption compared with mineral constituents of the soils.

In the soil pH with AS correlation, a non-significant and negative correlation was found between them. The availability of sulphur is also dominant at lower soil pH levels as compared to higher soil pH levels. On the other hand, organic carbon was significantly and positively correlated with AS. Although, the available soil sulphur content was found in higher per cent soil with increased soil organic levels.

The AS and AK were non-significantly and positively correlated and it was also found that at the very low level of AS and potassium were significantly and negatively correlated with each other (r=-0.143 and r=-0.135). Only a high S level of soil was non-significantly and positively correlated with AK soils. On the other hand, only low medium, and high medium levels of K were non-significantly and positively correlated with AS. This result indicated that some synergistic effect in terms of availability of these nutrients might be possible. This result corroborates the finding of Rietra et al. (2017).

## Conclusion

In conclusion, it was revealed that about 40% of the analyzed soil samples collected from the tea-growing region of Dooars were in a range below the optimum level of soil pH (4.50 to 5.50) deemed necessary for tea cultivation. Therefore, immediate necessary corrective measures should be adopted to improve soil pH and productivity in sustainable tea cultivation. The overall data of the study indicated that about 94% of soils of the Dooars region were between the satisfactory level and very good category of OC. However, to maintain the constant labile pool of OC in the soil, it is necessary to manage tea pruning litter properly to minimize the extra cost of organic manure application in the soil. It was also revealed that around 68.8% of the soils irrespective of the sub-districts were found to be deficient (≤100 mg kg^-1^) in the soil AK, whereas, only 31.2% of the soils were having >100 mg kg^-1^. Therefore, to improve and build-up of K in the tea-growing soils, the K fertilizers should be applied at the rate of 90 to 165 kg K_2_O ha^-1^ in four equal splits in a year based on the last pruning cycle yield to sustain the tea productivity. Around 43.25, 19.68 and 37.07% of the total analyzed soil samples irrespective of the sub-districts were low, medium, and high in soil AS. However, regular application of sulphur at 20-40 kg ha^-1^ with NPK fertilizers will be helpful to maintain crop productivity. In this study, it was found that most of the examined soils are low in pH index, and the AK and AS index were found to be medium in Dooars soils. Therefore, the adoption of appropriate soil management strategies would be helpful to improve the soil nutrient index.

## Data availability statement

The original contributions presented in the study are included in the article/**Supplementary Material**. Further inquiries can be directed to the corresponding authors.

## Author contributions

HM, and GT: Conceptualization, Investigation, Methodology, Formal analysis, Supervision, Validation, Writing -Original Draft. HM and JD: Writing -Original Draft. ABo, DD, and ABa: Data Curation, Visualization, Writing-Review and Editing. RP and MY: Data Curation, Statistical analysis, Visualization, Writing - Review and Editing. FR and SP: Formal analysis, Investigation, Validation, Writing -Original Draft. HM, DD, and TK: Conceptualization, Investigation, Methodology, Formal analysis, Supervision, Validation, Statistical analysis, Writing - Review and final Editing. All authors contributed to the article and approved the submitted version.
