# Supplementary material for: Sick or rich: Assessing the selected soil properties and fertility status across the tea-growing region of Dooars, West Bengal, India
Source: Front Plant Sci. 2022 Dec 20;13:1017145. doi: 10.3389/fpls.2022.1017145 (PMC9808038; doi:10.3389/fpls.2022.1017145)
Supplement: Supplementary file 1 [file DataSheet_1.docx]

**Appendix A. Supplementary Materials**

**Sick or Rich: Assessing the Selected Soil Properties and Fertility Status Across the Tea-growing Region of Dooars, West Bengal, India**

Harisadhan Malakar^1,*^, Gagan Timsina^2^, Jintu Dutta^1^, Arup Borgohain^3^, Diganta Deka^3^, Azariah Babu^1^, Ranjit Kumar Paul^4^, Md. Yeasin^4^, Feroze Hasan Rahman^5^, Saumik Panja^6^ Tanmoy Karak^3,$^

^1^Tocklai Tea Research Institute, Tea Research Association, Cinnamara, Jorhat, Assam 785008, India

^2^North Bank Advisory Centre, Thakurbari, Sonitpur, Assam-784503, India

^3^Upper Assam Advisory Centre, Tea Research Association, Dikom, Dibrugarh, Assam 786101, India

^4^Indian Agricultural Statistics Research Institute, New Delhi 110012, India

^5^ICAR-ATARI Kolkata, Salt Lake, Kolkata 700097, West Bengal, India

^6^University of California, Merced, 5200 N Lake Road, Merced, CA 95343

*Correspondence author: Tel.: +91 8902448855

Email addresses:[malakarhari@gmail.com](mailto:malakarhari@gmail.com); [h.malakar@tocklai.net](mailto:h.malakar@tocklai.net) (H. Malakar)

^$^Co-correspondence author: Tel.: +91 9435861567

Email addresses:[tanmay.karak@gmail.com](mailto:tanmay.karak@gmail.com); [t.karak@tocklai.net](mailto:t.karak@tocklai.net) (T. Karak)

**Supplementary Tables**

**Table S1 |**Information about sub-districts number of TEs in each sub-district, number of TEs included for the study, TEs cover in % and number of samples analyzed from each sub-district.

| **Sl. No.** | **Name of sub-districts** | **No. of gardens** | **No. of Gardens included** | **Garden cover in %** | **No. of soil samples analysed** |
| --- | --- | --- | --- | --- | --- |
| 1 | Binnaguri | 25 | 20 | 80.00 | 1604 (22.0)* |
| 2 | Chulsa | 35 | 17 | 48.57 | 808 (11.1) |
| 3 | Dalgaon | 30 | 16 | 53.33 | 579 (7.9) |
| 4 | Dam Dim | 34 | 26 | 76.47 | 1064 (14.6) |
| 5 | Jainti | 12 | 8 | 66.67 | 1364 (16.7) |
| 6 | Kalchini | 24 | 16 | 66.67 | 1220 (18.7) |
| 7 | Nagrakata | 15 | 12 | 80.00 | 661 (9.1) |
|  | **Overall** | **183** | **115** | **62.84** | **7300** |

*Values in parenthesis are the percent distribution of soil samples

**Table S2 |**Range and classification of soil parameters.

| **Soil parameters** | **Range** | **Classification** |
| --- | --- | --- |
| pH | ≤ 4.00 | Very Low |
|  | >4.00 - <4.25 | Moderately Low |
|  | >4.25 - <4.50 | Low |
|  | >4.50 - ≤5.50 | Optimum |
|  | >5.51 | High |
| Organic carbon (%) | ≤0.50 | Very Poor |
|  | >0.50 - ≤0.80 | Poor |
|  | >0.80 - ≤1.00 | Satisfactory |
|  | >1.00 - ≤1.50 | Moderately Good |
|  | >1.50 - ≤2.00 | Satisfactory Good |
|  | >2.00 - ≤2.50 | Good |
|  | >2.50 | Very Good |
| Available K as K_2_O (mg kg^-1^) | ≤ 40 | Very Low |
|  | >40 - ≤60 | Low |
|  | >60 - ≤80 | Low Medium |
|  | >80 - ≤100 | High Medium |
|  | > 100 | High |
| Available Sulphur (mg kg^-1^) | ≤ 20 | Very Low |
|  | >20 - ≤30 | Low |
|  | >30 - ≤40 | Medium |
|  | > 40 | High |

**Table S3 |**Nutrient Index categories of soil parameters.

| **Category** | **Parameters^*^** | | | |
| --- | --- | --- | --- | --- |
|  | **pH** | **OC (%)** | **AK (mg kg^-1^)** | **AS (mg kg^-1^)** |
| Low | <4.5 | <1.0 | <60 | <20 |
| Medium | 4.5-5.5 | 1.0‑1.5 | 60‑100 | 21‑40 |
| High | >5.50 | >1.5 | >100 | > 40 |
|  | **Nutrient Index** | **Range** | **Remarks** |  |
|  | **I** | <1.67 | Low |  |
|  | **II** | 1.67‑2.33 | Medium |  |
|  | **III** | > 2.33 | High |  |

^*^: OC- Organic carbon; AK- Available potassium and AS- Available sulphur

**Table S4****|** Number of soil samples and their percent contribution based on soil pH category in different Sub-districts of Dooars tea growing region, West Bengal, India.

| **Sub-districts** | **Category of soil pH with range** | | | | |
| --- | --- | --- | --- | --- | --- |
|  | **Very Low**  **(≤ 4.00)** | **Moderately**  **low**  **(>4.00 - ≤4.25)** | **Low**  **(>4.25 - ≤4.50)** | **Optimum**  **(>4.50-≤5.50)** | **High**  **(>5.50)** |
| Binnaguri | 176  (10.97)^*^ | 282  (17.58) | 289  (18.02) | 766  (47.76) | 91  (5.67) |
| Chulsa | 60  (7.43) | 148  (18.32) | 130  (16.09) | 458  (56.68) | 12  (1.49) |
| Dalgaon | 12  (2.07) | 37  (6.39) | 46  (7.94) | 383  (66.15) | 101  (17.44) |
| Dam Dim | 93  (8.74) | 175  (16.45) | 167  (15.70) | 604  (56.77) | 25  (2.35) |
| Jainti | 58  (4.75) | 120  (9.84) | 138  (11.31) | 759  (62.21) | 145  (11.89) |
| Kalchini | 182  (13.34) | 201  (14.74) | 206  (15.10) | 740  (54.25) | 35  (2.57) |
| Nagrakata | 171  (25.87) | 127  (19.21) | 90  (13.62) | 255  (38.58) | 18  (2.72) |
| Overall | 752  (10.30) | 1090  (14.93) | 1066  (14.60) | 3965  (54.32) | 427  (5.85) |

^*^: values in parentheses indicate the % of total samples in each sub-district

**Table S5|** Number of soil samples and their percent contribution based on soil organic carbon (OC) category in different Sub-districts of Dooars tea growing region, West Bengal, India.

| **Sub-districts** | **Category of soil OC (%) with range** | | | | | | |
| --- | --- | --- | --- | --- | --- | --- | --- |
|  | **Very poor**  **(≤0.50)** | **Poor**  **(>0.50 - ≤0.80)** | **Satisfactory (>0.80 - ≤1.00)** | **Moderately good**  **(>1.00 - ≤1.50)** | **Satisfactory good**  **(>1.50 - ≤2.00)** | **Good**  **(>2.00 - ≤2.50)** | **Very good**  **(>2.50)** |
| Binnaguri | 11  (0.69)^*^ | 53  (3.30) | 57  (3.55) | 298  (18.58) | 477  (29.74) | 456  (28.43) | 252  (15.71) |
| Chulsa | 8  (0.99) | 36  (4.46) | 31  (3.84) | 130  (16.09) | 196  (24.26) | 222  (27.48) | 185  (22.90) |
| Dalgaon | 14  (2.42) | 102  (17.62) | 142  (24.53) | 244  (42.14) | 61  (10.54) | 11  (1.90) | 5  (0.86) |
| Dam Dim | 8  (0.75) | 18  (1.69) | 21  (1.97) | 254  (23.87) | 398  (37.41) | 208  (19.55) | 157  (14.76) |
| Jainti | 6  (0.49) | 42  (3.44) | 90  (7.38) | 371  (30.41) | 335  (27.46) | 217  (17.79) | 159  (13.03) |
| Kalchini | 19  (1.39) | 118  (8.65) | 121  (8.87) | 554  (40.62) | 363  (26.61) | 133  (9.75) | 56  (4.11) |
| Nagrakata | 7  (1.06) | 8  (1.21) | 6  (0.91) | 68  (10.29) | 208  (31.47) | 234  (35.40) | 130  (19.67) |
| Overall | 73  (1.00) | 377  (5.16) | 468  (6.41) | 1919  (26.29) | 2038  (27.92) | 1481  (20.29) | 944  (12.93) |

^*^: values in parentheses indicate the % of total samples in each sub-district

**Table S6|** Number of soil samples and their percent contribution based on soil available potassium (AK) category in different Sub-districts of Dooars tea growing region, West Bengal, India.

| **Sub-districts** | **Category of soil AK (mg kg^-1^) with range** | | | | |
| --- | --- | --- | --- | --- | --- |
|  | **Very Low**  **(≤ 40)** | **Low**  **(>40 - ≤60)** | **Low Medium**  **(>60 - ≤80)** | **High Medium**  **(>80 - ≤100)** | **High**  **(>100)** |
| Binnaguri | 250  (15.59)^*^ | 367  (22.88) | 358  (22.32) | 210  (13.09) | 419  (26.12) |
| Chulsa | 85  (10.52) | 136  (16.83) | 159  (19.68) | 116  (14.36) | 312  (38.61) |
| Dalgaon | 130  (22.45) | 122  (21.07) | 121  (20.90) | 64  (11.05) | 142  (24.53) |
| Dam Dim | 99  (9.30) | 258  (24.25) | 246  (23.12) | 149  (14.00) | 312  (29.32) |
| Jainti | 180  (14.75) | 240  (19.67) | 267  (21.89) | 182  (14.92) | 351  (28.77) |
| Kalchini | 309  (22.65) | 372  (27.27) | 286  (20.97) | 133  (9.75) | 264  (19.35) |
| Nagrakata | 17  (2.57) | 32  (4.84) | 67  (10.14) | 67  (10.14) | 478  (72.31) |
| Overall | 1070  (14.66) | 1527  (20.92) | 1504  (20.60) | 921  (12.62) | 2278  (31.21) |

^*^: values in parentheses indicate the % of total samples in each sub-district

**Table S7 |** Number of soil samples and their percent contribution based on soil available sulphur (AS) category in different Sub-districts of Dooars tea growing region, West Bengal, India.

| **Sub-districts** | **Category of soil AS (mg kg^-1^) with range** | | | |
| --- | --- | --- | --- | --- |
|  | **Very Low**  **(≤ 20)** | **Low**  **(>20 - ≤30)** | **Medium**  **(>30 - ≤40)** | **High**  **(> 40)** |
| Binnaguri | 297  (18.52) | 279  (17.39) | 276  (17.21) | 752  (46.88) |
| Chulsa | 148  (18.32) | 175  (21.66) | 180  (22.28) | 305  (37.75) |
| Dalgaon | 118  (20.38) | 172  (29.71) | 134  (23.14) | 155  (26.77) |
| Dam Dim | 198  (18.61) | 181  (17.01) | 196  (18.42) | 489  (45.96) |
| Jainti | 373  (30.57) | 266  (21.80) | 246  (20.16) | 335  (27.46) |
| Kalchini | 343  (25.15) | 320  (23.46) | 272  (19.94) | 429  (31.45) |
| Nagrakata | 123  (18.61) | 164  (24.81) | 133  (20.12) | 241  (36.46) |
| Overall | 1600  (21.92) | 1557  (21.33) | 1437  (19.68) | 2706  (37.07) |

^*^: values in parentheses indicate the % of total samples in each sub-district

**Table S8 |**Relationship between different categories of soil pH and organic carbon (OC; %) contents of Dooars tea growing region, West Bengal, India.

| **OC⇨**  **pH**  **⇩** | **Very poor**  **(≤0.50)** | **Poor**  **(>0.50 - ≤0.80)** | **Satisfactory (>0.80 - ≤1.00)** | **Moderately Good**  **(>1.00 - ≤1.50)** | **Satisfactory Good**  **(>1.50 - ≤2.00)** | **Good**  **(>2.00 - ≤2.50)** | **Very Good**  **(>2.50)** | **Total** |
| --- | --- | --- | --- | --- | --- | --- | --- | --- |
| **Very Low (≤ 4.00)** | 0  (0.00)* | 6  (0.80) | 15  (1.99) | 10  (1.33) | 327  (43.48) | 101  (13.43) | 293  (38.96) | 752 |
| **Moderately Low**  **(>4.00 - ≤4.25)** | 3  (0.28) | 12  (1.10) | 20  (1.83) | 178  (16.33) | 315  (28.90) | 304  (27.89) | 258  (23.67) | 1090 |
| **Low(>4.25 - ≤4.50)** | 2  (0.19) | 17  (1.59) | 23  (2.16) | 236  (22.14) | 354  (33.21) | 268  (25.14) | 166  (15.57) | 1066 |
| **Optimum**  **(>4.50-≤5.50)** | 41  (1.03) | 268  (6.76) | 330  (8.32) | 1274  (32.13) | 1057  (26.66) | 664  (16.75) | 331  (8.35) | 3965 |
| **High (>5.50)** | 27  (6.32) | 74  (17.33) | 80  (18.74) | 130  (30.44) | 76  (17.80) | 24  (5.62) | 16  (3.75) | 427 |
| **Overall** | 73  (1.00) | 377  (5.16) | 468  (6.41) | 1828  (25.04) | 2129  (29.16) | 1361  (18.64) | 1064  (14.58) | 7300 |

^*^: values in parentheses indicate the % of total samples in each sub-district

**Table S9 |** Relationship between different categories of soil pH and available potassium (AK; mg kg^-1^) contents of Dooars tea growing region, West Bengal, India.

| **AK⇨**  **pH**  **⇩** | **Very Low**  **(≤ 40)** | **Low**  **(>40 - ≤60)** | **Low Medium**  **(>60 - ≤80)** | **High Medium**  **(>80 - ≤100)** | **High**  **(>100)** | **Total** |
| --- | --- | --- | --- | --- | --- | --- |
| **Very Low (≤ 4.00)** | 60  (7.98)^*^ | 96  (12.77) | 115  (15.29) | 101  (13.43) | 380  (50.53) | 752 |
| **Moderately Low(>4.00 - ≤4.25)** | 100  (9.17) | 200  (18.35) | 217  (19.91) | 143  (13.12) | 430  (39.45) | 1090 |
| **Low(>4.25 - ≤4.50)** | 131  (12.29) | 221  (20.73) | 246  (23.08) | 165  (15.48) | 303  (28.42) | 1066 |
| **Optimum(>4.50-≤5.50)** | 634  (15.99) | 904  (22.80) | 871  (21.97) | 474  (11.95) | 1082  (27.29) | 3965 |
| **High (>5.50)** | 145  (33.96) | 106  (24.82) | 55  (12.88) | 38  (8.90) | 83  (19.44) | 427 |
| **Overall** | 1070  (14.66) | 1527  (20.92) | 1504  (20.60) | 921  (12.62) | 2278  (31.21) | 7300 |

^*^: values in parentheses indicate the % of total samples in each sub-district

**Table S10 |** Relationship between different categories of soil pH and available sulphur (AS; mg kg^-1^) contents of Dooars tea growing region, West Bengal, India.

| **AS⇨**  **pH**  **⇩** | **Very Low**  **(≤ 20)** | **Low**  **(>20 - ≤30)** | **Medium**  **(>30- ≤40)** | **High**  **(> 40)** | **Total** |
| --- | --- | --- | --- | --- | --- |
| **Very Low (≤ 4.00)** | 156  (20.74) | 178  (23.67) | 162  (21.54) | 256  (34.04) | 752 |
| **Moderately Low(>4.00 - ≤4.25)** | 205  (18.81) | 218  (20.00) | 223  (20.46) | 444  (40.73) | 1090 |
| **Low(>4.25 - ≤4.50)** | 209  (19.61) | 214  (20.08) | 198  (18.57) | 445  (41.74) | 1066 |
| **Optimum(>4.50-≤5.50)** | 914  (23.05) | 866  (21.84) | 783  (19.75) | 1402  (35.36) | 3965 |
| **High (>5.50)** | 116  (27.17) | 81  (18.97) | 71  (16.63) | 159  (37.24) | 427 |
| **Overall** | 1600  (21.92) | 1557  (21.33) | 1437  (19.68) | 2706  (37.07) | 7300 |

^*^: values in parentheses indicate the % of total samples in each sub-district

**Table S11 |** Relationship between different categories of soil organic carbon (OC; %) and available potassium (AK; mg kg^-1^) contents of Dooars tea growing region, West Bengal, India.

| **AK⇨**  **OC**  **⇩** | **Very Low**  **(≤ 40)** | **Low**  **(>40 - ≤60)** | **Low Medium**  **(>60 - ≤80)** | **High Medium**  **(>80 - ≤100)** | **High**  **(>100)** | **Total** |
| --- | --- | --- | --- | --- | --- | --- |
| **Very poor (≤0.50)** | 37  (50.68)^*^ | 11  (15.07) | 14  (19.18) | 4  (5.48) | 7  (9.59) | 73 |
| **Poor (>0.50 - ≤0.80)** | 137  (36.34) | 104  (27.59) | 67  (17.77) | 22  (5.84) | 47  (12.47) | 377 |
| **Satisfactory (>0.80 - ≤1.00)** | 145  (30.98) | 113  (24.15) | 86  (18.38) | 59  (12.61) | 65  (13.89) | 468 |
| **Moderately Good (>1.00 - ≤1.50)** | 374  (19.49) | 470  (24.49) | 393  (20.48) | 218  (11.36) | 464  (24.18) | 1919 |
| **Satisfactory Good (>1.50 - ≤2.00)** | 206  (10.11) | 456  (22.37) | 443  (21.74) | 269  (13.20) | 664  (32.58) | 2038 |
| **Good (>2.00 - ≤2.50)** | 122  (8.24) | 260  (17.56) | 314  (21.20) | 205  (13.84) | 580  (39.16) | 1481 |
| **Very Good (>2.50)** | 49  (5.19) | 113  (11.97) | 187  (19.81) | 144  (15.25) | 451  (47.78) | 944 |
| **Overall** | 1070  (14.66) | 1527  (20.92) | 1504  (20.60) | 921  (12.62) | 2278  (31.21) | 7300 |

^*^: values in parentheses indicate the % of total samples in each sub-district

**Table S12. |** Relationship between different categories of soil organic carbon (OC; %) and available sulphur (AS; mg kg^-1^) contents of Dooars tea growing region, West Bengal, India.

| **AS⇨**  **OC**  **⇩** | **Very Low**  **(≤ 20)** | **Low**  **(>20 -≤30)** | **Medium**  **(>30- ≤40)** | **High**  **(> 40)** | **Total** |
| --- | --- | --- | --- | --- | --- |
| **Very poor (≤0.50)** | 39  (53.42)^*^ | 8  (10.96) | 3  (4.11) | 23  (31.51) | 73 |
| **Poor (>0.51 - ≤0.80)** | 83  (22.02) | 79  (20.95) | 77  (20.42) | 138  (36.60) | 377 |
| **Satisfactory (>0.81 - ≤1.00)** | 134  (28.63) | 113  (24.15) | 96  (20.51) | 125  (26.71) | 468 |
| **Moderately Good (>1.00 - ≤1.50)** | 479  (24.96) | 443  (23.08) | 363  (18.92) | 634  (33.04) | 1919 |
| **Satisfactory Good (>1.50 - ≤2.00)** | 434  (21.30) | 456  (22.37) | 419  (20.56) | 729  (35.77) | 2038 |
| **Good (>2.00 - ≤2.50)** | 246  (16.61) | 306  (20.66) | 320  (21.61) | 609  (41.12) | 1481 |
| **Very Good (>2.50)** | 185  (19.60) | 152  (16.10) | 159  (16.84) | 448  (47.46) | 944 |
| **Overall** | 1600  (21.92) | 1557  (21.33) | 1437  (19.68) | 2706  (37.07) | 7300 |

^*^: values in parentheses indicate the % of total samples in each sub-district

**Supplementary Figures**


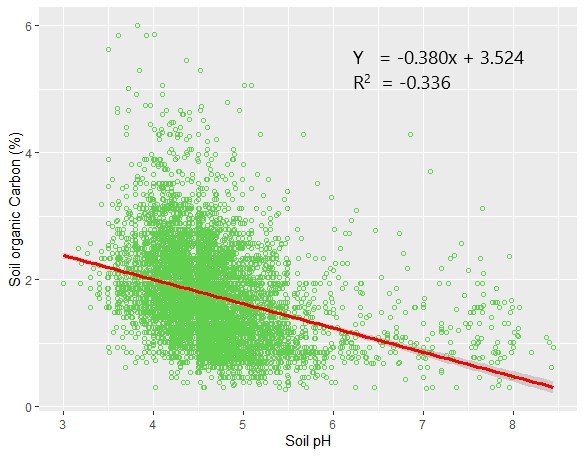


**Figure S1 |**Linear correlation between soil pH and organic carbon (%).


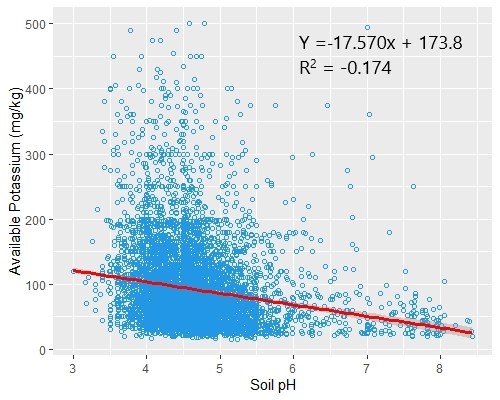


**Figure S2 |**Linear correlation between soil pH and available potassium (mg kg^-1^).


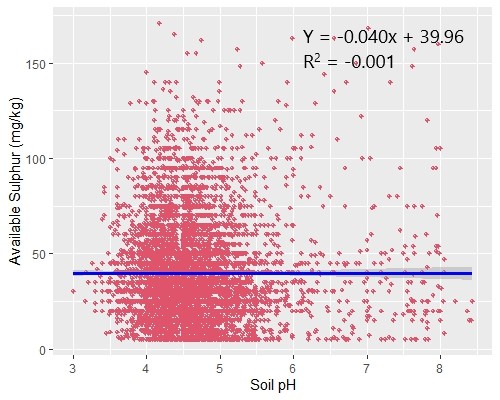


**Figure S3 |**Linear correlation between soil pH and available sulphur (mg kg^-1^).


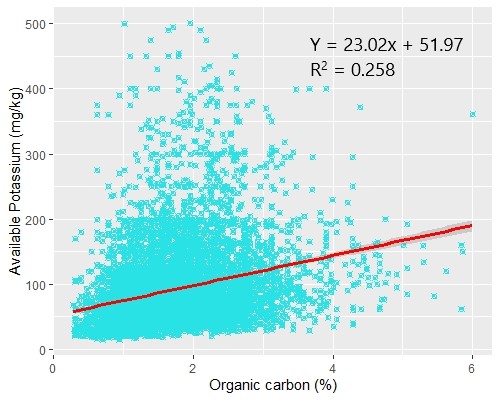


**Figure S4 |**Linear correlation between soil organic carbon (%) and available potassium (mg kg^-1^).


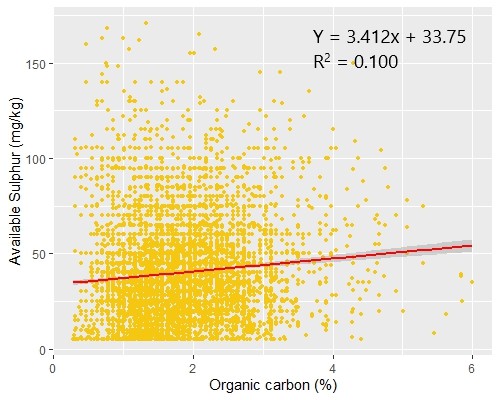
**Figure S5 |**Linear correlation between soil organic carbon (%) and available sulphur (mg kg^-1^).


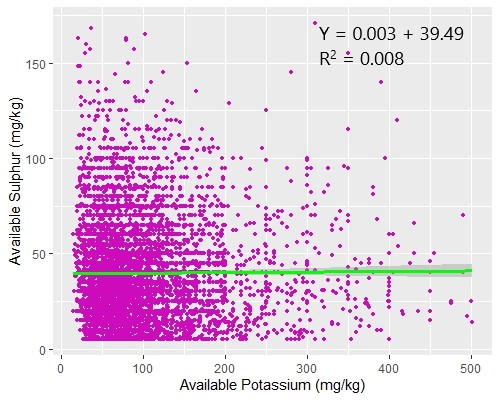


**Figure S6 |**Linear correlation between soil available potassium and sulphur (mg kg^-1^).

**
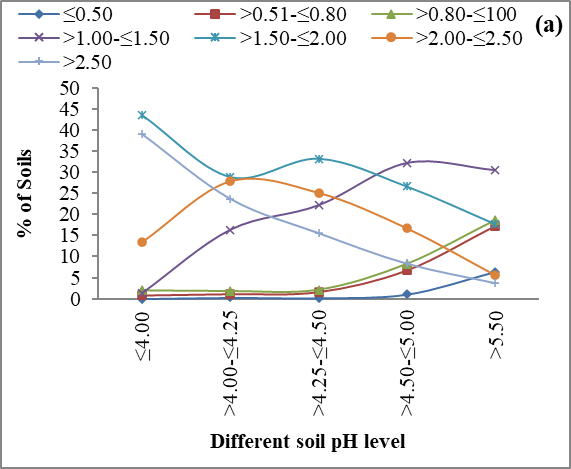

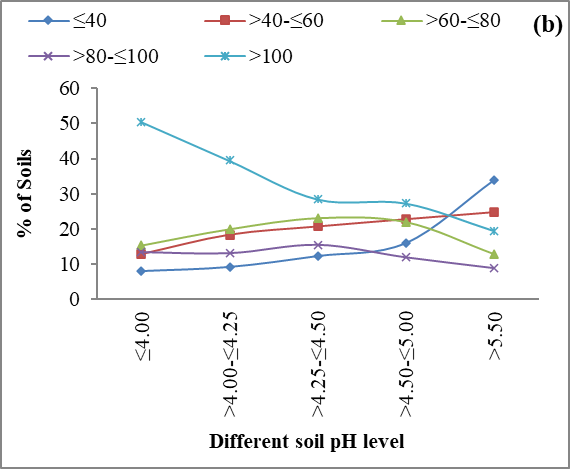

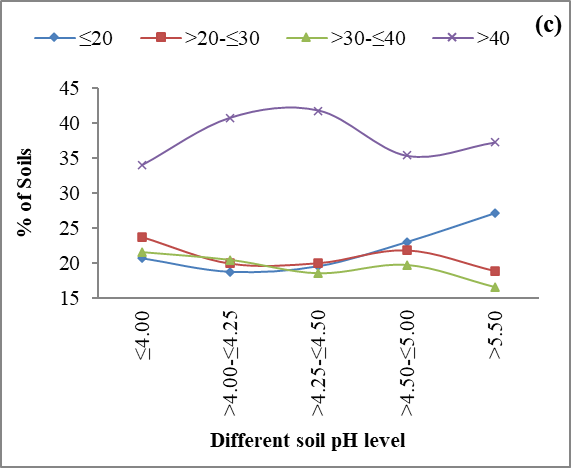

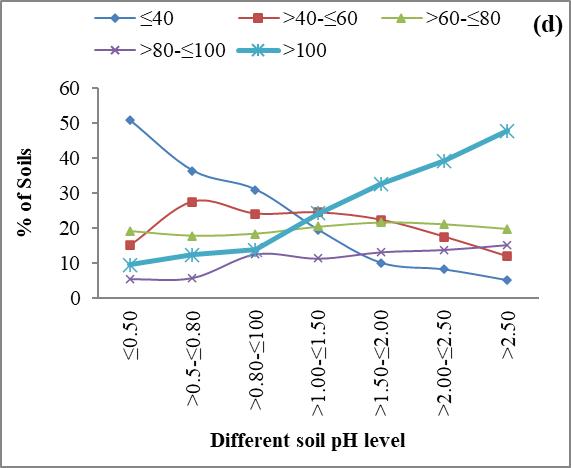

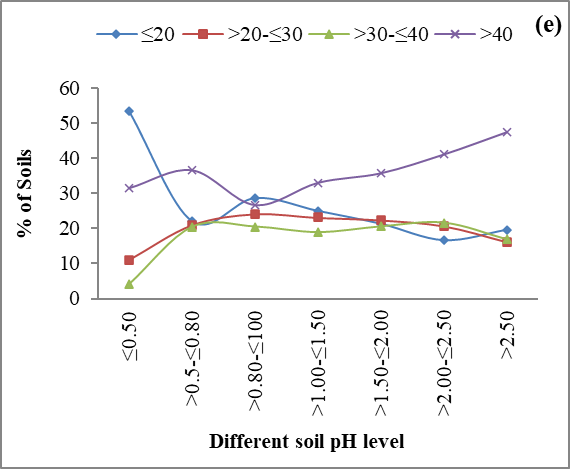
**

**Figure S7 |**Relation between (a) pH vs. organic carbon, (b) pH vs. available potassium, (c) pH vs. available sulphur, (d) organic carbon vs. available potassium and (e) organic carbon vs. available sulphur.
